# The dynamic nature of percolation on networks with triadic interactions

**DOI:** 10.1038/s41467-023-37019-5

**Published:** 2023-03-10

**Authors:** Hanlin Sun, Filippo Radicchi, Jürgen Kurths, Ginestra Bianconi

**Affiliations:** 1 School of Mathematical Sciences, Queen Mary University of London, London, E1 4NS UK; 2 Center for Complex Networks and Systems Research, Luddy School of Informatics, Computing, and Engineering, Indiana University, Bloomington, IN 47408 USA; 3 Potsdam Institute for Climate Impact Research, Potsdam, Germany; 4 Department of Physics, Humboldt University of Berlin, Berlin, Germany; 5 The Alan Turing Institute, The British Library, London, NW1 2DB UK

**Keywords:** Applied mathematics, Complex networks, Phase transitions and critical phenomena

## Abstract

Percolation establishes the connectivity of complex networks and is one of the most fundamental critical phenomena for the study of complex systems. On simple networks, percolation displays a second-order phase transition; on multiplex networks, the percolation transition can become discontinuous. However, little is known about percolation in networks with higher-order interactions. Here, we show that percolation can be turned into a fully fledged dynamical process when higher-order interactions are taken into account. By introducing signed triadic interactions, in which a node can regulate the interactions between two other nodes, we define triadic percolation. We uncover that in this paradigmatic model the connectivity of the network changes in time and that the order parameter undergoes a period doubling and a route to chaos. We provide a general theory for triadic percolation which accurately predicts the full phase diagram on random graphs as confirmed by extensive numerical simulations. We find that triadic percolation on real network topologies reveals a similar phenomenology. These results radically change our understanding of percolation and may be used to study complex systems in which the functional connectivity is changing in time dynamically and in a non-trivial way, such as in neural and climate networks.

## Abstract (web summary)

Triadic interactions are higher-order interactions relevant to many real complex systems. The authors develop a percolation theory for networks with triadic interactions and identify basic mechanisms for observing dynamical changes of the giant component such as the ones occurring in neuronal and climate networks.

## Introduction

Percolation^1–4^ is one of the most fundamental critical phenomena defined on networks. As such, it has attracted large interest in the literature^5–14^. Indeed by predicting the size of the giant component (GC) of a network when links are randomly damaged, percolation can be used for the establishment of the minimal requirements that a structural network should satisfy in order to support any type of interactive process. Despite the great success of percolation, ordinary percolation is unsuitable to describe real-world situations that occur in neuronal and climate networks when the connectivity of these networks changes in time.

Typically, the dynamics associated to percolation is the one of a cascading process where an initial failure propagates within a network possibly affecting its macroscopic connectedness. In the last decade, large scientific activity has been addressed to generalized percolation problems that capture cascades of failure events^5,15–20^ on multilayer networks^21–23^ where the damage propagates back and forth among the layers reaching a steady state at the end of the cascading process. In duplex networks, period-two oscillations can be observed in presence of competitive or antagonistic interactions^24–28^ among the different layers of the multiplex networks. However, this phenomenon seems to be restricted to duplex networks. Finally in damage and recovery models on multilayer networks^26,29,30^ aimed at getting insight for the robustness of complex critical infrastructures and financial systems, also more than two coexisting stable configurations of percolation have been observed.

An important question that arises from these works is whether percolation can capture more general time-dependent variations in the connectivity of a network. Here, we give a positive answer to this question and we show that higher-order interactions, and specifically triadic interactions, can turn percolation into a fully fledged dynamical process in which the order parameter undergoes period doubling and a route to chaos.

Higher-order networks are ubiquitous in nature^31–36^. Paradigmatic examples are the networks that describe brain activity, chemical reactions networks, and climate^37–41^. Higher-order interactions may profoundly change the physical properties of a dynamical process compared to those displayed by the same process occurring on a classic network of pairwise interactions. Examples include synchronization^42–45^, random walk dynamics^46^, contagion dynamics^47–52^ and game theory^53^. However little is known so far about percolation in presence of higher-order interactions^51,54–58^.

In this paper, we focus on a paradigmatic type of higher-order interactions named triadic interactions which occur when a node regulates the interaction between two other nodes. Regulation can be either positive, in the sense that the node facilitates the interaction, or negative, meaning that the regulator inhibits the interaction. Triadic interactions occur in ecosystems, where the competition between two species can be affected by the presence of a third species^59–61^. In neuronal networks, the interactions between neurons/glia is known to be triadic with glias modulating the synaptic interaction between neurons^62^. In climate networks of extreme rainfall events, triadic interactions can be used to explain the situations in which the network links are modulated by large-scale patterns, such as Rossby waves, which have a regulatory activity on climate inducing long-range synchronization of rainfall between Europe, Central Asia and even East Asia^40^. Finally in chemical reaction networks, generalized triadic interactions could model the action of enzymes as biological catalysts for biochemical reactions. While triadic interactions have received large attention in ecology and neuroscience, theoretical analyses of triadic interactions have investigated exclusively small-scale ecological systems^59–61^.

Here, we change perspective and study the role of triadic interactions in shaping macroscopic network properties. Specifically, we investigate how triadic interactions can change the critical and the dynamical properties of percolation. We combine percolation theory^1,3^ with the theory of dynamical systems^63–66^ to define triadic percolation, i.e., percolation in presence of signed triadic interactions. We show that in triadic percolation the GC of the network displays a highly non-trivial dynamics characterized by period doubling and a route to chaos. We use a general theory to demonstrate that the phase diagram of triadic percolation has fundamental differences with the phase diagram of ordinary percolation. While ordinary percolation displays a second-order phase transition, the phase diagram of triadic percolation is much richer and can be interpreted as an orbit diagram for the order parameter. Our theory is validated with extensive simulations on synthetic and real-world networks. These results reveal that in triadic percolation the GC of the network becomes a dynamical entity whose dynamics changes radically our understanding of percolation.

## Results

### Triadic interactions

**Fig. 1:**
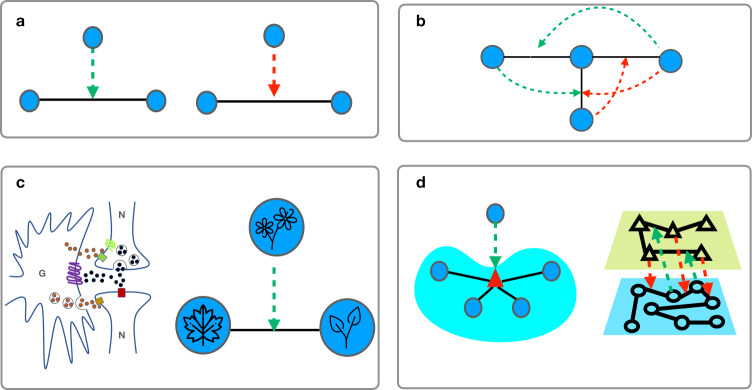
Triadic interactions. Triadic interactions occur when a node regulates the interactions between two other nodes. Triadic interactions can be signed with one node either favoring (green dashed link) or inhibiting (red dashed link) the interactions between the other to nodes (**a**). The simplest network including triadic interactions (**b**) is formed by a structural network between nodes and (solid line) structural links and a regulatory network including the regulatory interactions (dashed lines) between nodes and structural links. Examples of triadic interactions (**c**) include glias/neurons interactions and interactions between species in ecosystems. Triadic interactions can be extended to hypergraphs and multiplex networks (**d**). In hypergraphs the triadic interactions can regulate the presence or the activity of an hyperedge, in multiplex networks triadic interactions can be used to establish inter-layer interactions between nodes in one layers and links in the other layer. The plant icons are made by Freepik from www.flaticon.com.

Triadic interactions (see Fig. 1) are higher-order interactions between nodes and links. They occur when a node regulates the interaction between two other nodes. The regulation can be either positive, in the sense that the node facilitates the interaction, or negative, meaning that the regulator inhibits the interaction. For instance, the presence of a third species can enhance or inhibit the interaction between two species; also, the presence of a glia can favor or inhibit the synaptic interactions between two neurons. Triadic interactions can be added to a simple structural network. However, triadic interactions can also be introduced on top of an hypergraph, when one node regulates the strength of an hyperedge, or on top of multilayer networks, where triadic interactions represent inter-layer interactions between the nodes of one layer and the links of other layer. For instance, an enzyme is a node that can regulate an hyperedge (i.e., a reaction between chemicals); neural networks and networks of glias form instead two layers of a multiplex network interacting via triadic interactions.

Let us now formulate the simplest example of higher-order networks with triadic interactions. This higher-order network can be modeled as the composition of two networks: the structural network and the regulatory network which encodes triadic interactions. The structural network ${{{{{{{\mathcal{A}}}}}}}}=(V,\,E)$ is formed by the set of nodes *V* connected by the structural links in the set *E*. The regulatory network ${{{{{{{\mathcal{B}}}}}}}}=(V,\,E,\,W)$ is a bipartite, signed network between the set of nodes *V* of the structural network and the set of structural links *E*, with nodes in *V* regulating links in *E* on the basis of the regulatory interactions, either positive or negative, specified in the set *W*. Given a regulated link, a node at the end of the regulatory interaction is called positive regulator if the regulatory interaction is positive and negative regulator if the regulatory interaction is negative. Note that the sign is an attribute of the regulatory interaction and not of the node that acts as regulator.

In the following we will focus on percolation on this model of network with triadic interactions, however our results can be easily extended to hypergraphs and multiplex networks with triadic interactions as well.

### Triadic percolation

We define triadic percolation as the model in which the activity of the structural links is regulated by the triadic interactions and the activity of their regulator nodes. Conversely, the activity of the nodes is dictated by the connectivity of the network resulting after considering only the active links. In particular, we assume that the activity of nodes and links is changing in time leading to the triadic percolation process defined as follows. At time *t* = 0, every structural link is active with probability *p*_0_. We then iterate the following algorithm for each time step *t* ≥ 1:

- Step 1. Given the configuration of activity of the structural links at time *t* − 1, we define each node active if the node belongs to the GC of the structural network in which we consider only active links. The node is considered inactive otherwise.
- Step 2. Given the set of all active nodes obtained in step 1, we deactivate all the links that are connected at least to one active negative regulator node and/or that are not connected to any active positive regulator node. All the other links are deactivated with probability *q* = 1 − *p*.

Note that for *p* = *p*_0_ = 1 the model is deterministic. However, for *p* < 1 (and *p*_0_ < 1) the model is stochastic, i.e., the activity of the nodes does not uniquely define the activity of the links.

In the proposed triadic percolation, links can be dynamically turned on and off by the regulatory interactions. The model only makes minimal and justifiable assumptions while remaining general. The assumption that only nodes within the GC of the network are considered functioning/active is well accepted in the literature concerning network robustness^1,5^. Also, the regulatory rule chosen for deactivating the links is the minimal rule for treating both positive/negative regulations in a symmetric way: given suitable conditions the activation of a single positive regulator or the deactivation of a single negative regulator can turn the activity of a link on. Finally, the introduction of annealed stochastic effects, present for *p* < 1 (and *p*_0_ < 1), represents a simple way to account for the unavoidable randomness that can affect the activation/deactivation of the structural links in real scenarios.

**Fig. 2:**
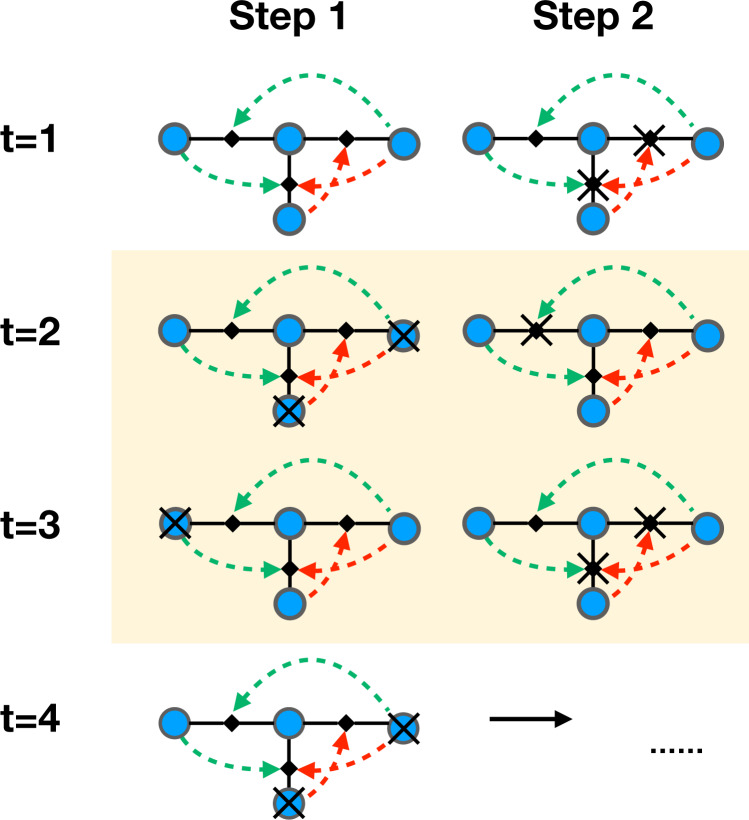
Sketch of triadic percolation. Solid lines represent structural links, dashed curves denote regulatory interactions (green stands for positive regulation, red for negative). Blue filled circles indicate structural nodes, black diamonds indicate triadic interactions. For simplicity, we consider the deterministic bond-percolation model for *p* = *p*_0_ = 1. At each stage *t* of the dynamics, bond percolation is applied to the network, and then the effect of the regulatory activity is established. The illustration shows how the dynamics sets into a periodic pattern with the giant component of the network “blinking” in time. The periodic pattern is highlighted in yellow. At time *t* = 1, all links are active and all nodes are part of the giant component (GC). Their regulatory activity causes some links to become inactive (crossed links in the figure). As a consequence, at time *t* = 2, some nodes are no longer part of the GC and become inactive (crossed nodes in the figure). However, this change leads to changes in the activity of some links, which in turn affect the activity of the nodes at time *t* = 3, 4, etc. The final configuration reached at time *t* = 3 is identical to one observed at the end of stage *t* = 1. Due to the determinism of the model, the pattern repeats with period *T* = 2. The relative size *R* of the GC oscillation switches between 2/4 and 3/4. For an example of more complex dynamical behavior see Supplementary Movie.

Triadic percolation can lead to a highly non-trivial dynamics of the network connectivity. For instance Fig. 2 illustrates the phenomenon of network “blinking” with nodes of the network turning on and off periodically to form GCs of different size. As we will see, this dynamics emerges at the bifurcation transition indicating the onset of the period-two oscillations of the order parameter, but oscillations of longer period and also chaos is observed depending on the model’s parameters.

### Theory of triadic percolation

Here we establish the theory for triadic percolation that is able to predict the phase diagram of the model on random networks with triadic interactions.

We assume that the structural network ${{{{{{{\mathcal{A}}}}}}}}$ is given and contains *N* nodes and 〈*k*〉*N*/2 structural links, with 〈*k*〉 indicating the average degree of the network. We consider structural networks given by individual instances of the configuration model. To this end, we first generate degree sequences by selecting random variables from the degree distribution *π*(*k*). We denote with *k*_*i*_ the structural degree of node *i*.

To generate the regulatory network ${{{{{{{\mathcal{B}}}}}}}}$, we assume that every node *i* has associated two degree values, namely the number of positive regulatory interactions ${\kappa }_{i}^{+}$, and the number of negative regulatory interactions ${\kappa }_{i}^{-}$. For simplicity we consider the case in which both ${\kappa }_{i}^{+}$ and ${\kappa }_{i}^{-}$ are chosen independently of the structural degree *k*_*i*_ (see the SI for the extension to the correlated case). Each structural link *ℓ* is assigned the degrees ${\hat{\kappa }}_{\ell }^{+}$ and ${\hat{\kappa }}_{\ell }^{-}$ indicating the number of positive regulators and the number of negative regulators, respectively. In particular, nodes’ degrees are extracted at random from the distribution *P*(*κ*^+^, *κ*^−^), and links’ degrees are randomly extracted from the distribution $\hat{P}({\hat{\kappa }}^{+},\,{\hat{\kappa }}^{-})$ here taken to be uncorrelated so that $\hat{P}({\hat{\kappa }}^{+},\,{\hat{\kappa }}^{-})={\hat{P}}_{+}({\hat{\kappa }}^{+}){\hat{P}}_{-}({\hat{\kappa }}^{-})$. Once degrees have been assigned to nodes and links, we establish the existence of a positive (+) or negative (−) regulatory interaction between the structural link *ℓ* and the node *i* with probability:

1 $${p}_{\ell,i}^{\pm }=\frac{{\kappa }_{i}^{\pm }{\hat{\kappa }}_{\ell }^{\pm }}{\langle {\kappa }^{\pm }\rangle N},$$

where 〈*κ*^±^〉 denotes the average of *κ* over all the nodes of the network. In the creation of regulatory interactions, we allow any pair (*ℓ*, *i*) to be connected either by a positive of by a negative regulatory interaction but not by both. Note that as long as the network ${{{{{{{\mathcal{B}}}}}}}}$ is large and sparse the latter condition is not inducing significant correlations.

Let us now combine the theory of percolation with the theory of dynamical systems to derive the phase diagram of the considered uncorrelated scenario. Let us define *S*^(*t*)^ as the probability that a node at the endpoint of a random structural link of the network ${{{{{{{\mathcal{A}}}}}}}}$ is in the GC at time *t*. Moreover, let us indicate by *R*^(*t*)^ the fraction of nodes in the GC at time *t* (or equivalently the probability that a node at the end of a regulatory link is active). Finally, ${p}_{L}^{(t-1)}$ is the probability that a random structural link is active at time *t*. By putting ${p}_{L}^{(0)}={p}_{0}$ indicating the probability that structural links are active at time *t* = 0, we have that for *t* > 0, as long as the network is locally tree like, *S*^(*t*)^, *R*^(*t*)^ and ${p}_{L}^{(t)}$ are updated as:

2 $${S}^{(t)}	=1-{G}_{1}\left(1-{S}^{(t)}{p}_{L}^{(t-1)}\right),\\ {R}^{(t)}	=1-{G}_{0}\left(1-{S}^{(t)}{p}_{L}^{(t-1)}\right),\\ {p}_{L}^{(t)}	=p{G}_{0}^{-}(1-{R}^{(t)})\left[1-{G}_{0}^{+}\left(1-{R}^{(t)}\right)\right],$$

where the first two equations implement Step 1, i.e., a bond-percolation model^1^ where links are retained with probability ${p}_{L}^{(t-1)}$, and the third equation implements Step 2, i.e., the regulation of the links. Here the generating functions *G*_0_(*x*), *G*_1_(*x*) and ${{G}_{0}}^{\pm }(x)$ are given by:

3 $$\begin{array}{c}{G}_{0}(x)=\mathop{\sum}\limits_{k}\pi (k){x}^{k},\quad {G}_{1}(x)=\mathop{\sum}\limits_{k}\pi (k)\frac{k}{\langle k\rangle }{x}^{k-1},\\ {{G}_{0}}^{\pm }(x)=\mathop{\sum}\limits_{{\kappa }_{\pm }}{\hat{P}}_{\!\!\pm }({\hat{\kappa }}^{\pm }){x}^{{\hat{\kappa }}^{\pm }}.\end{array}$$

Equation (2) for the percolation model regulated by triadic interactions can be formally written as the map^65^:

4 $${R}^{(t)}=f\left({p}_{L}^{(t-1)}\right),\quad {p}_{L}^{(t)}={g}_{p}\left({R}^{(t)}\right),$$

which can be further reduced to a unidimensional map *R*^(*t*)^ = *h*(*R*^(*t*−1)^). The previous set of equations lead to the theoretical prediction for triadic percolation defined on structural networks generated according to the configuration model. This solution are of mean-field nature: while triadic percolation dynamics has many interacting degrees of freedom given by the activity of each node and each link, and is characterized by a stochastic dynamics for *p* < 1, Eq. (2) [or equivalently the map Eq. (4)] involves only three/two variables and are deterministic. As we will see, despite this approximations, the proposed theoretical approach provides a very accurate prediction of the behavior of triadic percolation.

**Fig. 3:**
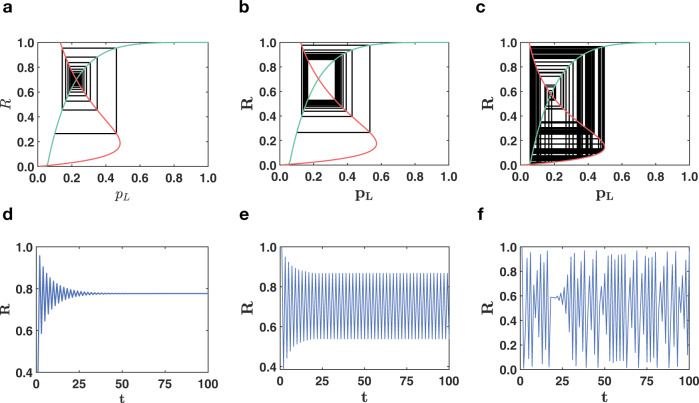
Time dependence of the order parameter of triadic percolation. In triadic percolation, the order parameter *R* can have non-trivial dynamics. Here we demonstrate with theory and simulations the non-trivial dynamics of *R* for parameters values in which the dynamics reaches a steady state (**a**, **d**), period-two oscillations (**b**, **e**) and a chaotic dynamics (**c**, **f**). This behavior is predicted by the theory which can be schematically represented by cobweb plots (**a**–**c**) corresponding to the map Eq. (4) with the function *f* indicated in green and the function *g*_*p*_ in red. Results of Monte Carlo simulations for *R* as a function of time *t* (**d**–**f**) are in excellent agreement (MC) with the theory. The structural network has a power-law degree distribution *π*(*k*) ~ *k*^−*γ*^, with minimum degree *m* = 4, maximum degree *K* = 100, and degree exponent *γ* = 2.5. The degrees ${\hat{\kappa }}^{+}$ and ${\hat{\kappa }}^{-}$ of the regulatory network obey Poisson distributions with average *c*^+^ and *c*^−^. The links are activated with probability *p* = 0.8. The parameters *c*^+^, *c*^−^ are *c*^+^ = 10, *c*^−^ = 1.8 (**a**, **d**), *c*^+^ = 10, *c*^−^ = 2.1 (**b**, **e**). The MC simulations are performed an networks of *N* = 10^4^ nodes.

**Fig. 4:**
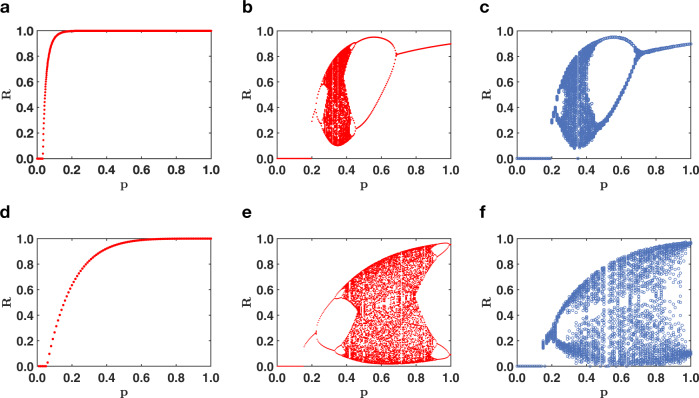
Phase diagram of triadic percolation on Poisson and scale-free structural networks. The phase diagram of triadic percolation (**b**, **c**, **e**, **f**) is radically different from the phase diagram of ordinary percolation (**a**, **d**) for both Poisson (**a**–**c**) and scale-free structural networks (**d**–**f**). Ordinary percolation reveals a second-order phase transition (theoretical prediction, **a**, **d**) while the phase diagram of triadic percolation reveals that the order parameter *R* displays period doubling and a route to chaos (**b**, **c**, **e**, **f**). The theoretical predictions of the phase diagram obtained from Eq. (2) are in very good agreements with the phase diagram obtained from extensive Monte Carlo (MC) simulations (**e**, **f**). In **a**–**c**, the structural network is Poisson with average degree *c* = 30; the regulatory network is also Poisson with averages *c*^+^ = 1.8 and *c*^−^ = 2.5. In **d**, **e**, the scale-free structural network has degree exponent *γ* = 2.5, minimum degree *m* = 4 and maximum degree *K* = 100; the regulatory network is Poisson with *c*^+^ = 10 and *c*^−^ = 2.8. The MC simulations are obtained from networks of size *N* = 2 × 10^5^ (**e**) and *N* = 10^4^ (**f**). Here points represent all *R* values observed in the time range 150 ≤ *t* ≤ 200.

In presence of negative interactions, triadic percolation displays a time-dependent order parameter, given by the active fraction of nodes *R*^(*t*)^. The order parameter *R*^(*t*)^ undergoes a period doubling and a route to chaos in the universality class of the logistic map for structural networks with arbitrary degree distribution *π*(*k*) and regulatory connectivity generated by Poisson distributions $P({\hat{\kappa }}^{\pm })$ (see SI and Supplementary Figs. 1–5 for details). Triadic percolation has a very rich dynamical nature and displays the emergence of both “blinking” oscillations and chaotic patterns of the giant component (see Fig. 3). “Blinking” refers to the intermittent switching on and off of two or more sets of nodes which leads to periodic oscillations of the order parameter. Chaos implies that at each time a different set and number of nodes is activated. The map defined by Eq. (4) allows us to generate the cobweb of the dynamical process. Theoretical predictions display excellent agreement with extensive simulations of the model (see Fig. 3). The combination of negative and positive regulatory interactions present in triadic percolation leads to a much richer phase diagram than the one of ordinary percolation in absence of triadic interactions (see Fig. 4). The phase diagram of triadic percolation is found by monitoring the relative size *R* of the GC as a function of the parameter *p* indicating the probability that a link is active when all the regulatory conditions allowing the link to be active are satisfied. Clearly from Fig. 4, we see that while in absence of triadic interactions the transition is second-order; when signed positive and negative regulatory interactions are taken into account, the phase diagram of percolation becomes an orbit diagram. In particular, Eq. (2) predicts that the order parameter undergoes a period doubling and a route to chaos irrespective of the degree distribution of the structural network. Theoretical predictions are well matched by results of numerical simulations (see Fig. 4). Our theory allows to well approximate the dynamical behavior of triadic percolation for random Poisson and scale-free structural networks (see Supplementary Information (SI) and Supplementary Figs. 6–11 for a discussion about the effect of the structural degree distribution on the phase diagram of triadic percolation).

**Fig. 5:**
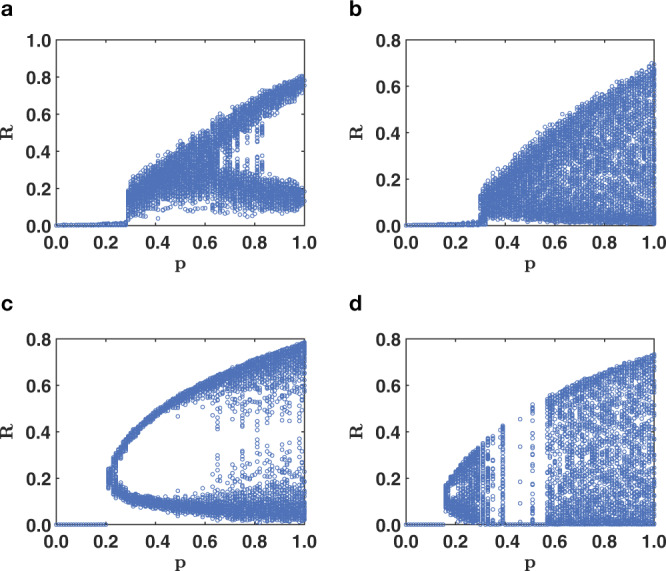
Phase diagram of triadic percolation for real-world structural network topologies. The phase diagram of triadic interaction displaying the fraction of nodes *R* in the GC as a function of *p* is shown for real-world structural networks obtained from the repository^67^: the mouse brain network (**a**, **b**) the Human bio grid network (**c**, **d**). The phase diagrams are obtained by MC simulations with Poisson regulatory networks with parameters *c*^+^ = 20, *c*^−^ = 2 (**a**), *c*^+^ = 20, *c*^−^ = 4 (**b**); *c*^+^ = 20, *c*^−^ = 4 (**c**). *c*^+^ = 20, *c*^−^ = 6 (**d**). All orbit diagrams are obtained with an initial condition ${p}_{L}^{(0)}=0.1$.

Results of numerical simulations denote a rich dynamical behavior of the model also if structural networks are taken from the real-world. In particular, we consider real-world structural networks constructed from empirical data collected in the repository of ref. ^67^, and we combine these real structural networks with synthetic regulatory networks capturing the triadic interactions. In Fig. 5, we show that also for these topologies the phase diagram reveals non-trivial dynamics with some regimes of (noisy) oscillations and some regimes of chaotic dynamics of the order parameter (for more information about these datasets see Supplementary Table 1 and Supplementary Fig. 12).

**Fig. 6:**
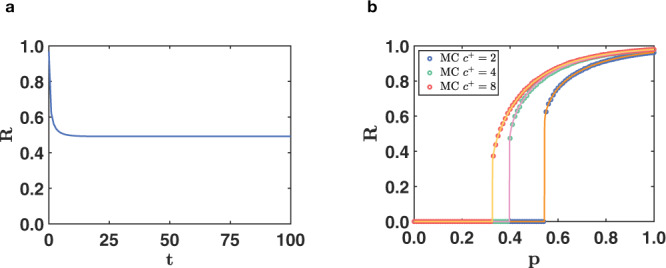
Triadic percolation in absence of negative triadic interactions. In absence of negative triadic interactions the order parameter *R* of triadic percolations always reaches a stationary state for sufficiently long times (**a**). Moreover the phase diagram, indicating the stationary solution of *R* as a function of *p* displays a discontinuous hybrid transition (**b**). In **b**, the results obtained from MC simulations (symbols) over networks of *N* = 10^4^ nodes are compared to theoretical expectations (solid curves). In both plots the Poisson structural network has average degree *c* = 4, the Poisson regulatory network including exclusively positive regulations has average degree *c*^+^. In **a**, the results re shown for *p* = 0.4 and *c*^+^ = 4. In Supplementary Table 1 and in Supplementary Fig. 11 we provide more information about these datasets.

In absence of negative triadic interactions, when all regulatory interactions are positive, the dynamics always reaches a stationary point independent of time. In Fig. 6a we show a typical time-series for *R*^(*t*)^ where it is apparent that *R* reaches a stationary limit *R*^(*t*)^ = *R*^⋆^, where *R*^⋆^ is independent of time. Moreover in Fig. 6b we also display the dependence of this stationary state with *p*, i.e., *R* = *R*^⋆^(*p*). The agreement between theoretical predictions and results of numerical simulations is excellent. Interestingly, the order parameter *R* displays a discontinuous hybrid phase transition as a function of *p* showing that positive triadic interactions induce discontinuous hybrid percolation in higher-order networks (see Fig. 6 and the SI for the analytical derivation of this result).

In order to exclude that the observed chaotic behavior of triadic percolation is an artefact of the particular choice of the dynamics, we consider also a version of the model with time-delayed regulatory interactions, where each regulatory link is assigned a time delay *τ* and Step 2 of triadic percolation is replaced by:

- Step 2′. Given the set of all active nodes obtained in Step 1, each structural link is deactivated:
  - if none of its positive regulators is active at time at *t* − *τ*;
  - if at least one of its negative regulators is active at time *t* − *τ*;
  - if the structural link is not deactivated according the conditions (a) and (b), it can still be deactivated by stochastic events which occur with probability *q* = 1 − *p*.

**Fig. 7:**
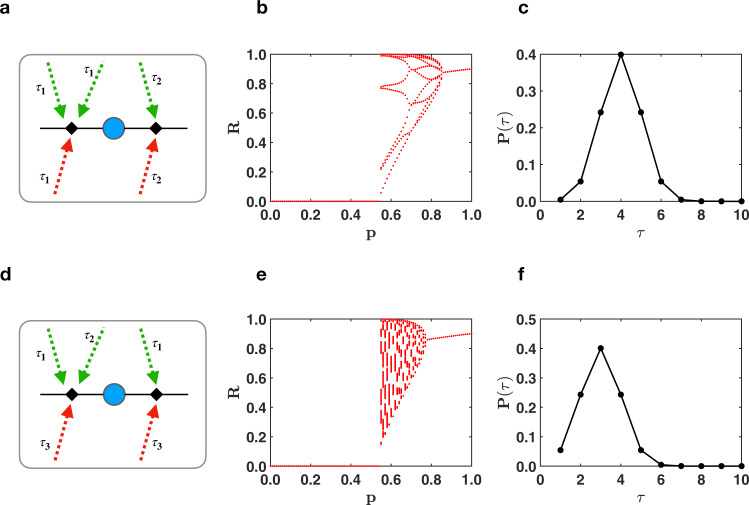
Triadic percolation with time delays. Model 1 and Model 2 of triadic percolation with delays are illustrated in **a** and **d**, respectively. The corresponding phase diagram for structural Poisson network are shown in **b** for Model 1 and in **d** for Model 2. The orbit diagrams in **b** and **e** are obtained for the $\tilde{p}(\tau )$ distribution of delays shown in **c** and **f**, respectively. The two orbit diagrams are obtained from the same structural and regulatory network. The structural network is a Poisson network with an average degree *c* = 50 and the regulator network has Poisson degree distribution with average degree *c*^+^ = 10 and *c*^−^ = 3.3.

We consider two models of triadic percolation with time delay which depend on the choice of the probability distribution for time delays of regulatory links (see the illustration of the models in Fig. 7):

- [Model 1] each structural link is regulated by regulatory links associated to the same time delay *τ*, with the time delay *τ* being drawn from the distribution $\tilde{p}(\tau )$;
- [Model 2] each regulatory link is associated to a time delay drawn independently from the distribution $\tilde{p}(\tau )$.

Note that both models reduce to triadic percolation without delays when $\tilde{p}(\tau )={\delta }_{\tau,1}$ where *δ*_*x*,*y*_ indicates the Kronecker delta. Interestingly both models lead to a route to chaos also in presence of a non-trivial distribution of time delays, although the universality class might be different from the one of the logistic map (see Fig. 7). This finding demonstrates that the route to chaos observed in triadic percolation is a robust feature of the triadic-percolation model. Finally we note that triadic percolation might be suitably generalized also to node percolation leading also in this case to a route to chaos for the order parameter *R* (see SI and Supplementary Fig. 13 for details about this generalization of triadic percolation).

## Discussion

A combination of positive and negative interactions is known to affect statistical mechanics problems in non-trivial ways^68,69^. For instance the introduction of signed interactions in the Ising model change dramatically the phase diagram of the model and gives rise to spin glasses with a complex free-energy landscape which display a very different structure of equilibrium configurations with respect to the Ising model. Here we combine the theory of percolation with the theory of dynamical systems and we show that positive and negative regulatory triadic interactions can turn percolation into a fully fledged dynamical process where the order parameter undergoes a period doubling and a route to chaos. This implies that, although the underlying structural network remains the same, links and nodes can be activated and deactivated in time leading to a very non-trivial dynamics of the giant component of the network which can “blink” among few possible connectivity configurations or change in time in a chaotic way. This implies that triadic percolation is radically different from standard percolation in which the activity of the links is not dynamically regulated and for each value of *p* characterizing the probability that links are active, the order parameter takes only a single value. This significant effect of triadic interaction on percolation is captured by the striking difference between the phase diagram of triadic percolation and the phase diagram of standard percolation. While standard percolation leads to a second-order phase transition, the phase diagram of triadic percolation, as long as negative regulatory interactions are included, becomes an orbit diagram. In absence of negative regulatory interactions, triadic percolation has an order parameter that always reaches a steady state and the phase diagram displays a discontinuous hybrid phase transition. Our conclusions are based on a general theory giving very accurate predictions although being of a mean-field nature and on extensive simulations performed on synthetic as well as real-world network topologies.

The model can be modified in different ways to address the needs for specific real systems. For instance the approach can be applied to other generalized network structures such as hypergraphs and multiplex networks. Moreover the regulatory rules adopted can be modified. Finally, the approach can be extended to situations in which the nodes sustain a more complex dynamics.

These results radically change our understanding of percolation and can be used to shed light on real systems in which the functional connectivity of the network is strongly dependent on time as in neuronal and brain networks and in climate. A particularly promising future direction is to apply this theoretical framework to modeling extreme rainfall events. This could lead to a substantial improvement of their forecasting.

## Supplementary information

Supplementary Information

Description of Additional Supplementary Files

Supplementary Movie

## Data Availability

All the datasets used in this study are available on the public repository ref. ^67^.

## Code availability

The codes used in this study are available at the GitHub repository Triadic percolation, https://zenodo.org/record/7651480; 10.5281/zenodo.7651480.
