# Supplementary material for: The dynamic nature of percolation on networks with triadic interactions
Source: Nat Commun. 2023 Mar 10;14:1308. doi: 10.1038/s41467-023-37019-5 (PMC9998640; doi:10.1038/s41467-023-37019-5)
Supplement: Supplementary file 1 — Supplementary Information [file 41467_2023_37019_MOESM1_ESM.pdf]

Supplementary Information
"The dynamic nature of percolation on networks with triadic interactions"

Hanlin Sun[1], Filippo Radicchi[2], Jürgen Kurths[3,4] & Ginestra Bianconi[1,5]

[1]*School of Mathematical Sciences, Queen Mary University of London, London E1 4NS, United Kingdom*

[2]*Center for Complex Networks and Systems Research, Luddy School of Informatics, Computing, and Engineering, Indiana University, Bloomington, 47408, USA*

[3]*Potsdam Institute for Climate Impact Research, Potsdam, Germany*

[4]*Department of Physics, Humboldt University of Berlin, Berlin, Germany*

[5]*The Alan Turing Institute, The British Library, London NW1 2DB, United Kingdom*

1 Triadic percolation for correlated structural and regulatory networks

General theoretical framework- In this section we extend the theoretical approach described in the main text in order to treat also the case in which the structural degree k of a node can be correlated with its regulatory degrees κ^+ and κ^-.

In this case the random higher-order network with triadic interactions is characterized by a joint degree distribution $\tilde{P}(k, \kappa^+, \kappa^-)$ and by the degree distributions $\hat{P}_\pm(\hat{\kappa}^\pm)$. The distribution $\tilde{P}(k, \kappa^+, \kappa^-)$ indicates the probability that a random node has structural degree k and regulatory degrees κ^+ and κ^-. The distributions $\hat{P}_\pm(\hat{\kappa}^\pm)$ indicates the probability that a random link has $\hat{\kappa}^+$ or $\hat{\kappa}^-$ regulatory interactions, respectively.

Let us define $S^{(t)}$ as the probability that a node at the endpoint of a random structural link of network $\mathcal{A}$ is in the giant component (GC) at time t. Let us define $\hat{S}^{(t)\pm}$ as the probability that a node regulating (positively $+$ or negatively $-$) a random structural link is in the GC at time t. Let us define with $p_L^{(t)}$ the probability that a random structural link is active at time t. By putting $p_L^{(0)} = p_0$ indicating the probability that structural links are active at time $t = 0$, we have that for all $t > 0$, as long as the network is locally tree-like, $S^{(t)}$, $\hat{S}^{(t)\pm}$ and $p_L^{(t)}$ are updated as

$$\begin{aligned} S^{(t)} &= 1 - G_1\left(1 - S^{(t)} p_L^{(t-1)}\right), \\ \hat{S}^{(t)\pm} &= 1 - \mathcal{G}^\pm(1 - S^{(t)} p_L^{(t-1)}), \\ p_L^{(t)} &= p G_0^-(1 - \hat{S}^{(t)-}) \left[1 - G_0^+(1 - \hat{S}^{(t)+})\right], \end{aligned} \tag{1}$$

where

$$
\begin{aligned}
G_1(x) &= \sum_{k,\kappa^+,\kappa^-} \tilde{P}(k,\kappa^+,\kappa^-)\frac{k}{\langle k\rangle}x^{k-1}, \\
G_0{}^{\pm}(x) &= \sum_{\kappa_\pm} \hat{P}_\pm(\hat{\kappa}^\pm)x^{\hat{\kappa}^\pm}, \\
\mathcal{G}^{\pm}(x) &= \sum_{k,\kappa^+,\kappa^-} \tilde{P}(k,\kappa^+,\kappa^-)\frac{\kappa^\pm}{\langle \kappa^\pm\rangle}x^{k}.
\end{aligned}
\tag{2}
$$

The probability that a node is in the GC is given by

$$
R^{(t)} = 1 - G_0\left(1 - S^{(t)}p_L^{(t-1)}\right), \tag{3}
$$

where

$$
G_0(x) = \sum_{k,\kappa^+,\kappa^-} \tilde{P}(k,\kappa^+,\kappa^-)x^k. \tag{4}
$$

The stationary solution and the onset of its instability- The equations for triadic percolation can be formally written as a map [1]:

$$
\hat{S}^{(t)\pm} = f^{\pm}(p_L^{(t-1)}), \quad p_L^{(t)} = g_p(\hat{S}^{(t),+}, \hat{S}^{(t)-}), \tag{5}
$$

whose stationary fixed point $\hat{S}^{\star\pm}, p_L^{\star}$ satisfies

$$
\hat{S}^{\star\pm} = f^{\pm}(g_p(\hat{S}^{\star,+}, \hat{S}^{\star-})), \tag{6}
$$

or equivalently

$$
p_L^{\star} = g_p(f^{+}(p_L^{\star}), f^{-}(p_L^{\star})). \tag{7}
$$

The stationary solution becomes unstable when

$$
|J| = 1, \tag{8}
$$

where

$$J = \left. \frac{\mathrm{d}g_p(f^+(p_L), f^-(p_L))}{\mathrm{d}p_L} \right|_{p_L = p_L^\star} . \tag{9}$$

As we will show in the next paragraphs, there are two major types of instability. The first type of instability is observed when $J = 1$ and leads to discontinuous hybrid transitions. This type of instability is observed for instance for triadic percolation in absence of negative interactions. The second type of instability is achieved instead when $J = -1$ and this leads typically to the onset of period 2 oscillations of the order parameter $R^{(t)}$ of percolation.

Note that the stability of the periodic oscillations of the order parameter can be studied in an analogous way by investigating the stability of the map iterated for a number of times equal to the period of the oscillation under study. However, we leave this analysis to later works.

Limiting case of uncorrelated structural and regulatory degrees of the nodes- If we assume that the structural and regulatory degrees of the nodes are uncorrelated, we can then write

$$\tilde{P}(k, \kappa^+, \kappa^-) = \pi(k) P(\kappa^+, \kappa^-). \tag{10}$$

The equations (1) do simplify as $\mathcal{G}^+(x) = \mathcal{G}^-(x)$ and the phase diagram is independent of the degree distribution $P(\kappa^+, \kappa^-)$. Therefore in this limit we recover Eq. (2) of the main text that we

repeat here for completeness, i.e.,

$$S^{(t)} = 1 - G_1\left(1 - S^{(t)} p_L^{(t-1)}\right), \tag{11}$$

$$R^{(t)} = 1 - G_0\left(1 - S^{(t)} p_L^{(t-1)}\right), \tag{12}$$

$$p_L^{(t)} = pG_0^{-}(1 - R^{(t)})\left[1 - G_0^{+}\left(1 - R^{(t)}\right)\right]. \tag{13}$$

Here, we have used the simplified definition of the generating functions given by

$$\begin{aligned} G_0(x) &= \sum_k \pi(k) x^k, \\ G_1(x) &= \sum_k \pi(k) \frac{k}{\langle k \rangle} x^{k-1}, \\ G_0{}^{\pm}(x) &= \sum_{\kappa_\pm} \hat{P}_\pm(\hat{\kappa}^\pm) x^{\hat{\kappa}^\pm}. \end{aligned} \tag{14}$$

As noted in the main text, Eq. (11)-(13) for the percolation model regulated by triadic interactions can be formally written as the map [1]

$$R^{(t)} = f\left(p_L^{(t-1)}\right), \quad p_L^{(t)} = g_p\left(R^{(t)}\right), \tag{15}$$

or combining these two equations as the map

$$R^{(t)} = h_p\left(R^{(t-1)}\right) = f\left(g_p\left(R^{(t-1)}\right)\right). \tag{16}$$

The stationary solution $R^{(t)} = R^\star$ of this map obeys the equation

$$R^\star = h_p(R^\star). \tag{17}$$

This stationary solution becomes unstable as soon as

$$|J| = 1, \tag{18}$$

where

$$J = h'_p(R^\star) = \left.\frac{df}{dp_L}\right|_{p_L=p_L^\star} \left.\frac{dg_p}{dR}\right|_{R=R^\star}. \tag{19}$$

Interestingly, while the value $J = 1$ indicates a discontinuous and hybrid transition, the value $J = -1$ indicates the onset of period-2 oscillations.

Let us here show that the discontinuous transition observed for $J = 1$ is actually hybrid. To this end we indicate with p_c the value of p for which $J = 1$ is satisfied and we consider small variations $\delta p = p - p_c \ll 1$. We indicate the corresponding change in the stationary solution $R^\star$ with $\delta R = R^\star(p) - R^\star(p_c) \ll 1$. Since both $R^\star(p)$ and $R^\star(p_c) = R_c$ satisfy the stationary Eq. (17), assuming without loss of generality that $h_p(R^\star)$ is twice differentiable at $R^\star(p_c) = R_c > 0$ we can expand this latter equation in δp and $\delta R^\star$ obtaining

$$\delta R = h'_{p_c}(R_c)\delta R + \frac{1}{2}h''_{p_c}(R_c)(\delta R)^2 + \frac{\partial h_{p_c}(R_c)}{\partial p}\delta p. \tag{20}$$

Since $h'_{p_c}(R_c) = 1$ this equation reduces to

$$\frac{1}{2}h''_{p_c}(R_c)(\delta R)^2 + \frac{\partial h_{p_c}(R_c)}{\partial p}\delta p = 0, \tag{21}$$

from which it is immediate to derive the scaling $\delta R \propto (\delta p)^{1/2}$ as long as $h''_{p_c}(R_c)$ and $\partial h_{p_c}(R_c)/\partial p$ have finite values and opposite sign. Therefore we have shown that

$$R^\star(p) - R_c \propto (p - p_c)^{1/2}, \tag{22}$$

which establishes that the discontinuous transition is hybrid.

In order to provide evidence and motivation of the general result that $J = -1$ indicates the onset of period two oscillations in the next section, we will show a concrete example. For

the moment, let us observe that the explicit expression of J given by Eq. (19) implies that since $df/dp_L \geq 0$ the onset of the period-2 oscillations can take place only if $g_p(R)$ has a negative slope. As a consequence of this we conclude that period-2 oscillations can occur only if negative regulatory interactions are present.

In Fig. $S1$ we show the cobweb when only positive regulatory interactions are present, i.e. when Eq. (13) is substituted by

$$p_L^{(t)} \quad = \quad p\left[1 - G_0^{+}\left(1 - R^{(t)}\right)\right]. \tag{23}$$

In Fig. $S2$ we show the cobweb when only the negative regulatory interactions are present and positive interactions do not play a role, i.e. when Eq. (13) is substituted by

$$p_L^{(t)} \quad = \quad pG_0^{-}(1 - R^{(t)}). \tag{24}$$

For examples of the cobweb when both positive and negative interactions are present and relevant see Fig. 3 of the main text.

2 Triadic percolation for uncorrelated Poisson structural network

Triadic percolation on uncorrelated Poisson structural networks In this section we investigate the instability of the stationary solution in the case of a Poisson structural network of average degree c in which the structural and the regulatory degrees of the nodes are uncorrelated, i.e.,

$$\tilde{P}(k, \kappa^{+}, \kappa^{-}) = \pi(k)P(\kappa^{+}, \kappa^{-}), \tag{25}$$

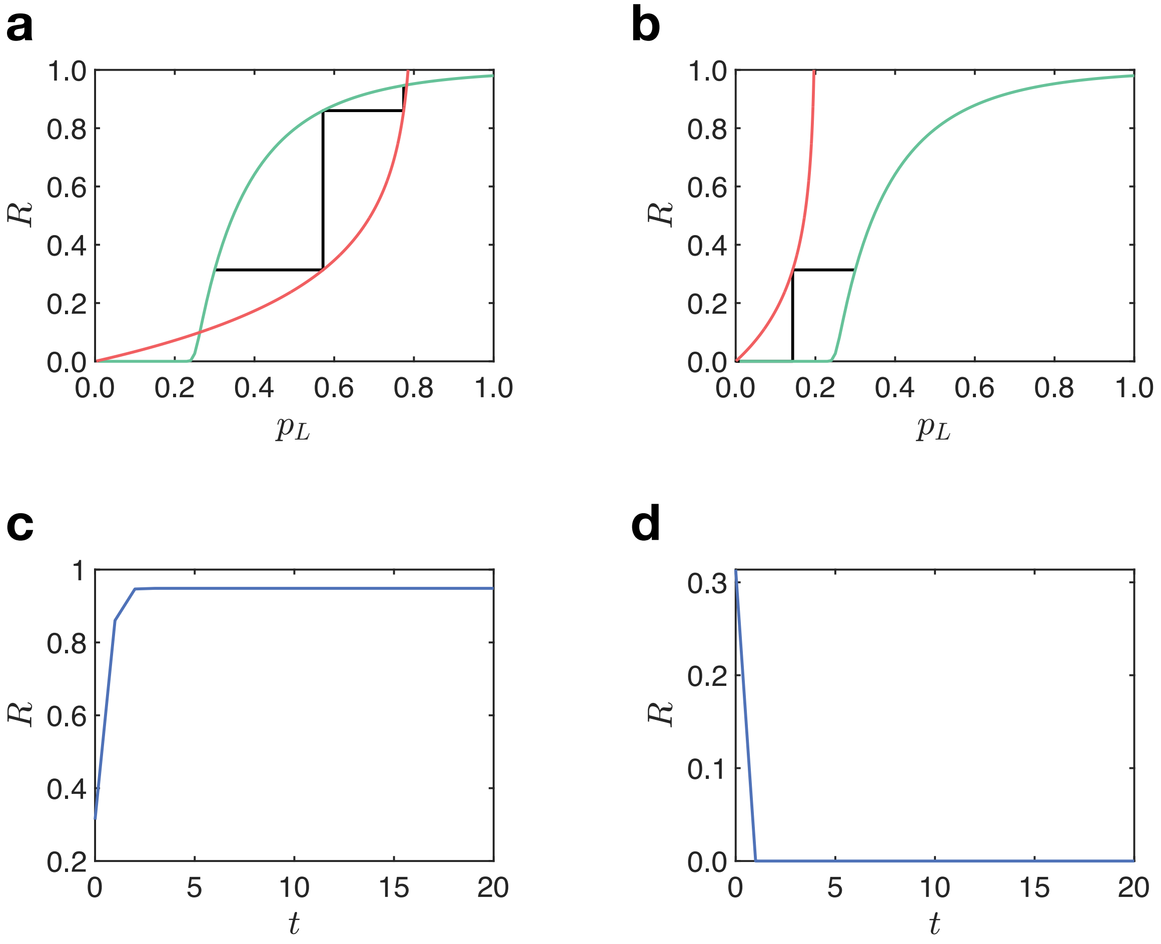

Figure S1: The theoretical cobweb plot (panel (a), (b)) and the corresponding dependence of the order parameter R on time t (panel (c), (d)) is shown when regulatory interactions are exclusively positive. The structural network is a Poisson network with average degree $c = 4$, and Poisson distribution $\hat{P}_{\pm}(\hat{\kappa}_{\pm})$ with average degrees $c^{+} = 4$ and $c^{-} = 0$ respectively. In panel (a) and (c) $p = 0.8 > p_c$; in panel (b) and (d) $p = 0.2 < p_c$. The results are obtained with an initial condition $p_L^{(0)} = 0.3$.

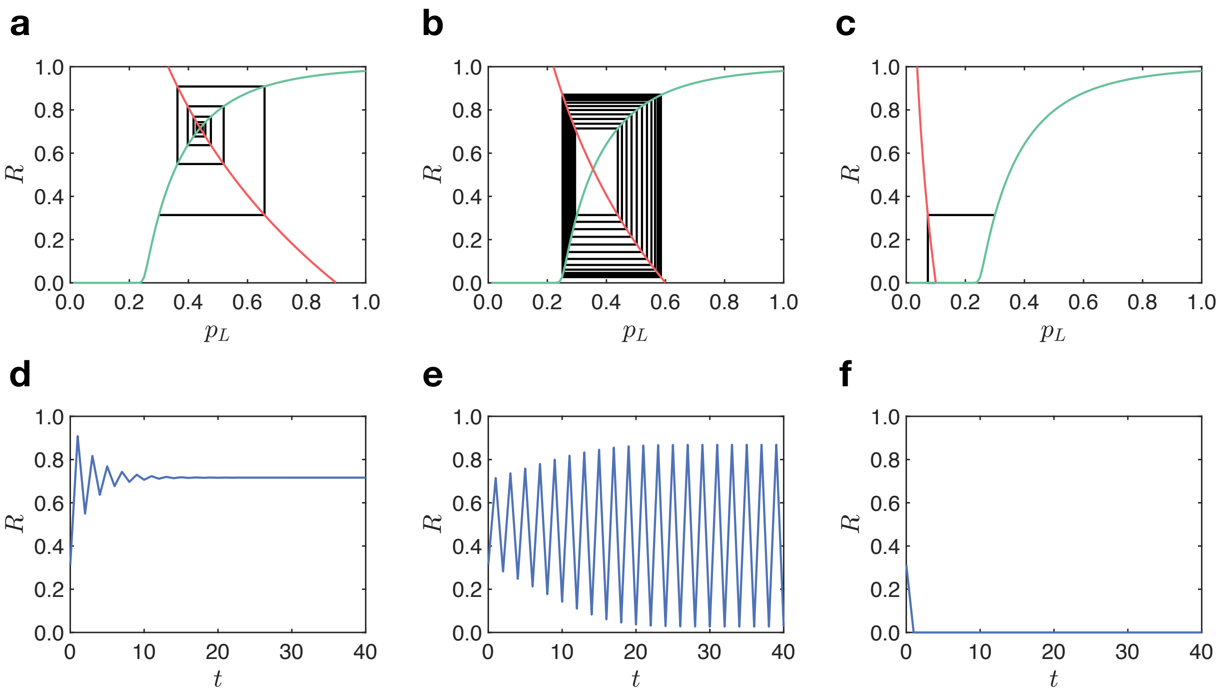

Figure S2: The theoretical cobweb plot (panel (a), (b), (c)) and the corresponding dependence of the order parameter R on time t (panel (d), (e), (f)) is shown when regulatory interactions are exclusively negative. The structural network is a Poisson network with average degree $c = 4$, and Poisson distribution $\hat{P}_{\pm}(\hat{\kappa}_{\pm})$ with average degrees $c^{+} = 4$ and $c^{+} = \infty$ respectively. In panel (a) and (d) $p = 0.9$; in panel (b) and (e) $p = 0.6$; in panel (c) and (f) $p = 0.1$. The results are obtained with an initial condition $p_L^{(0)} = 0.3$.

with

$$\pi(k) \;\; = \;\; \frac{1}{k!} c^k e^{-c}. \tag{26}$$

Additionally we assume that $\hat{\kappa}^+$ and $\hat{\kappa}^-$ are drawn from Poisson distributions with average degree c^+ and c^- respectively, i.e.,

$$\hat{P}_\pm(\hat{\kappa}^\pm) = \frac{1}{\hat{\kappa}^\pm!}(c^\pm)^{\hat{\kappa}^\pm} e^{-c^\pm}. \tag{27}$$

Eq. ((12) and Eq. ((13) for the triadic percolation reduces to

$$\begin{aligned} R^{(t)} &= 1 - e^{-cp_L^{(t-1)} R^{(t)}} \\ p_L^{(t)} &= p\left(1 - e^{-c^+ R^{(t)}}\right) e^{-c^- R^{(t)}}. \end{aligned} \tag{28}$$

The first equation can be expressed as a map between $p_L^{(t-1)}$ and $R^{(t)}$, while the second equation can be expressed as a map between $R^{(t)}$ and $p_L^{(t)}$, i.e.,

$$R^{(t)} = f\left(p_L^{(t-1)}\right), \quad p_L^{(t)} = g_p\left(R^{(t)}\right). \tag{29}$$

Both equations can be combined in the single map

$$R^{(t)} = h_p\left(R^{(t-1)}\right) = f\left(g_p\left(R^{(t-1)}\right)\right). \tag{30}$$

Onset of the instability of the stationary solutions- Triadic percolation on a structural Poisson network and a Poisson regulatory network with average degrees c^+ and c^- for positive and negative regulatory interactions, admits a stationary steady state when Eq. (28) have the solution $R^{(t)} = R^\star, p_L^{(t)} = p_L^\star$, where $R^\star$ and $p_L^\star$ satisfy

$$\begin{aligned} R^\star &= 1 - e^{-cp_L^\star R^\star}, \\ p_L^\star &= p\left(1 - e^{-c^+ R^\star}\right) e^{-c^- R^\star}. \end{aligned} \tag{31}$$

These equations can be expressed as a single equation

$$R^\star = h_p(R^\star) = f(g_p(R^\star)), \tag{32}$$

where the functions $h_p(R)$, $f(p_L)$ and $g_p(R)$ have the same definition as in the previous paragraph. The stationary solution becomes unstable for

$$|J| = |h_p'(R^\star)| = \left|\frac{df(g_p(R^\star))}{dR^\star}\right| = \left|f'(p_L^\star)g_p'(R^\star)\right| = 1, \tag{33}$$

where f' and g' are given by

$$\begin{aligned} f'(p_L^\star) &= -\frac{cR^\star}{cp_L^\star - e^{cp_L^\star R^\star}}, \\ g_p'(R^\star) &= p(c^+ + c^-)e^{-(c^- + c^+)R^\star} - c^- p e^{-c^- R^\star}. \end{aligned} \tag{34}$$

Solving Eq. (31) and Eq. (33) numerically when $J = 1$ we find the critical manifold of discontinuous hybrid transitions and when $J = -1$ we find the manifold for the onset of period-2 oscillations of the order parameter $R^{(t)}$.

In Fig. $S3$ we show graphically the difference between the two types of possible instabilities of the stationary solution $R^\star = h(R^\star)$. When $J = h'(R^\star) = 1$ one observes the discontinuous emergence of a non-zero stationary solution $R^\star > 0$. When $J = h'(R^\star) = -1$ we observe the onset of period-2 oscillations of the order parameter satisfying the map $R^{(t)} = h(R^{(t-1)})$.

In Fig. $S4$ we show the obtained critical manifolds for the onset of period two oscillations of the order parameters and for the onset of discontinuous hybrid transitions. Note that in for any given structural and regulatory networks the critical point of the onset of the discontinuous hybrid

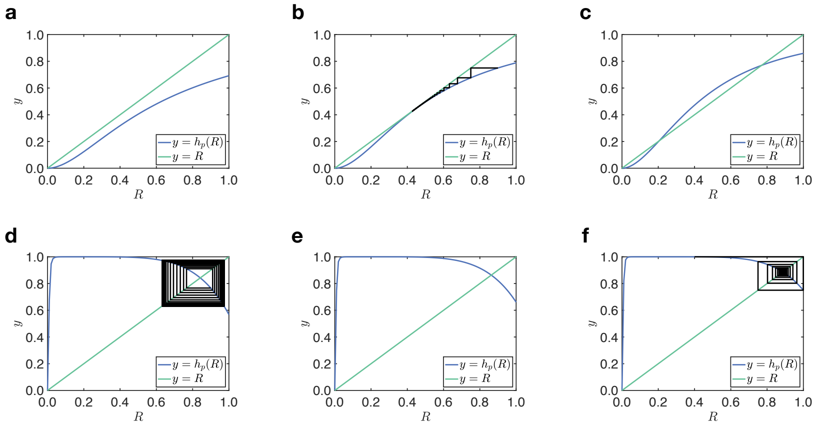

Figure S3: The figures show the two different modalities for the onset of the instability of the stable solution of the iterative map $R^{(t)} = h_p(R^{(t-1)})$ for a Poisson network with triadic interactions corresponding to the crossing of the curves $y = h_p(R)$ and $y = R$. In panels (a),(b),and (c) we show the emergence of the discontinuous transition at $p = 0.392$ (panel (b)) on a Poisson network with average degree $c = 4$, and Poisson distribution s $\hat{P}^{\pm}(\hat{\kappa}^{\pm})$ with average degrees $c^{+} = 4$ and $c^{-} = 0$ respectively. Panels (a) and (c) show the functions $y = h_p(R)$ and $y = R$ for $p = 0.30$ (below the transition) and $p = 0.50$ (above the transition). Note that in panel (b) the function $y = h(R)$ and the function $y = R$ are tangent to each other at their non-trivial intersection indicating that the non-trivial solution disappears as soon as $p < 0.392$. In panel (d),(e),(f) we show the emergence of 2-cycle at $p = 0.665$ (panel (e)) for a Poisson network with average degree $c = 30$, and Poisson distributions $\hat{P}^{\pm}(\hat{\kappa}^{\pm})$ with average degree $c^{+} = 10$ and $c^{-} = 2.5$ respectively. Panels (d) and (f) show the functions $y = h(R)$ and $y = R$ for $p = 0.60$ (below the transition) and $p = 0.8$ (above the transition) respectively. Note that in panel (e) the function $y = h_p(R)$ displays a derivative -1 leading to the emergence of the 2-limit cycle observed for $p \leq 0.665$. The relative cobweb are shown only for panels (b), (d) and (f) to improve the readability of the figure.

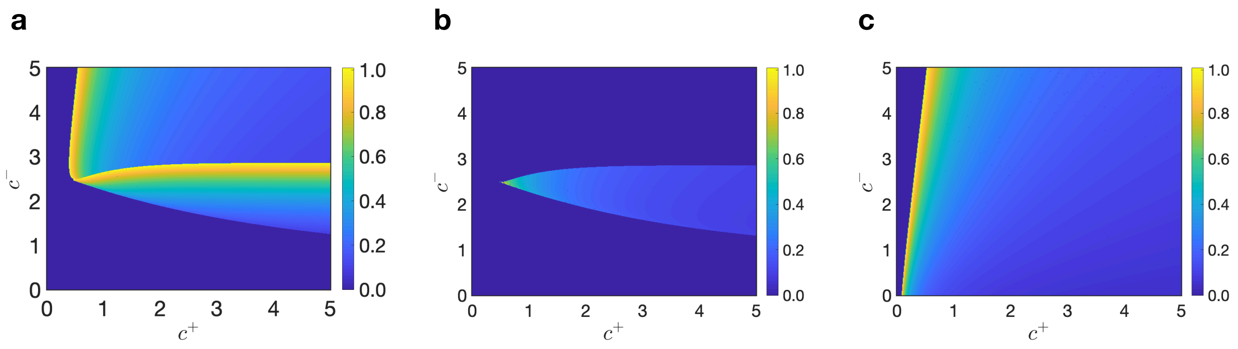

Figure S4: The upper critical point p_c^u (panel a) and the lower critical point p_c^l (panel b) determining the onset of period-2 oscillations are plotted plane (c^+, c^-). Panel c represents the critical point p_c at which the discontinuous hybrid transition is observed in the plane (c^+, c^-). In all panels the structural network has Poisson degree distribution with degree $c = 30$ the regulatory network has also a Poisson degree distribution with c^+ and c^- indicating the average positive and negative degree respectively.

transition is unique, if such transition exist. However the onset of the period two oscillations can occur for different values of p. In Fig. $S4$ we plot exclusively the larger and the smaller critical points for the onset of period two oscillations if they exist.

3 Universality class of the route to chaos of triadic percolation

In the previous sections we have studied triadic percolation in different settings and we have shown that the process can undergo a period doubling transition. In this section we demonstrate that triadic percolation undergoes a route to chaos in the universality class of the logistic map as long as the structural and regulatory degrees are uncorrelated and the distributions $P(\hat{\kappa}^{\pm})$ are Poisson.

Logistic map universality class Triadic percolation can be captured at the mean-field level by a map

$$R^{(t)} = h(R^{(t-1)}) \tag{35}$$

determining the relative size $R^{(t)}$ of the giant component at time t, given the relative size $R^{(t-1)}$ of the giant component at time $t-1$. Examples of these maps obtained from uncorrelated structural Poisson networks are shown in Figure $S5$. Here we show that this map is in the universality class of the logistic map. In order to show that, according to Feigenbaum classic result[2], it is enough to demonstrate that the function $h(R)$ is unimodal, i.e., has a single maximum at $R = R^\star$, and that close to its maximum, i.e., for $|R - R^\star| \ll 1$, the function $h(R)$ has a quadratic approximation, with

$$h(R) \simeq h(R^\star) + \frac{1}{2}h''(R^\star)(R - R^\star)^2. \tag{36}$$

To demonstrate this scaling of the function $h(R)$ close to its maximum we provide here the explicit expression of the derivative $dR^{(t)}/dR^{(t-1)}$ in terms of $R^{(t-1)}$ and $R^{(t)} = h(R^{(t-1)})$.

Our starting point will be the formulation of triadic percolation for uncorrelated structural and regulatory degrees of the nodes dictated by the Eqs.(11),(12) and (13), which we rewrite here for completeness,

$$\begin{aligned} S^{(t)} &= 1 - G_1\left(1 - p_L^{(t-1)} S^{(t)}\right) = F_1\left(p_L^{(t-1)}, S^{(t)}\right), && (37)\\ R^{(t)} &= 1 - G_0\left(1 - p_L^{(t-1)} S^{(t)}\right) = F_2\left(p_L^{(t-1)}, S^{(t)}\right), && (38)\\ p_L^{(t)} &= pG_0^-\left(1 - R^{(t)}\right)\left[1 - G_0^+\left(1 - R^{(t)}\right)\right] = F_3\left(R^{(t)}\right), && (39) \end{aligned}$$

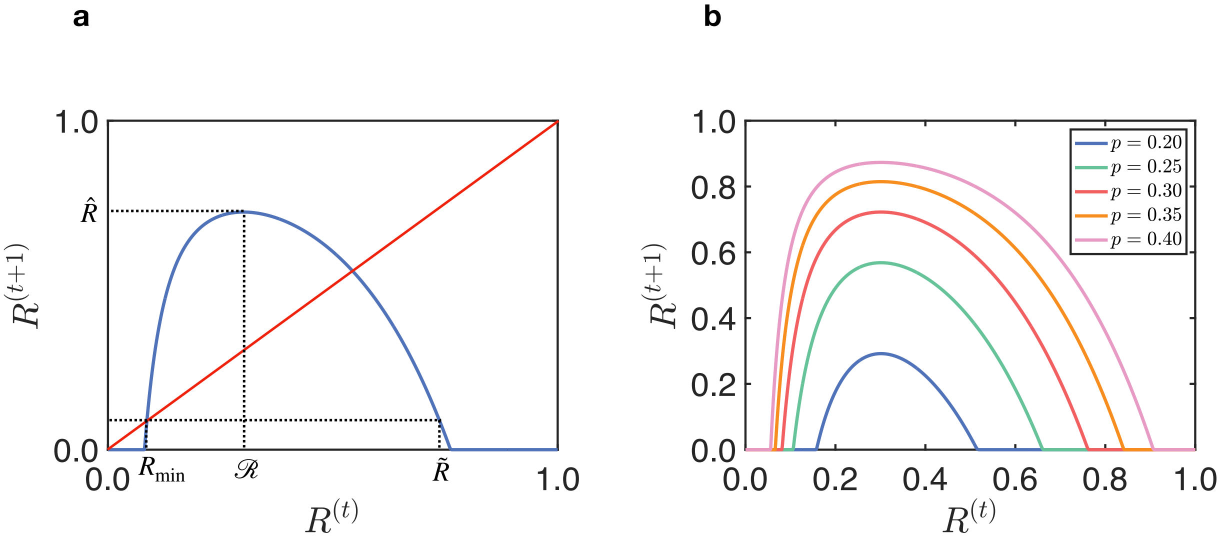

Figure S5: **Example of the maps capturing triadic percolation.** Panel (a) shows the map $R^{(t)} = h(R^{(t-1)})$ (in blue) obtained for $p = 0.3$ in the case of a Poisson structural network and uncorrelated regulatory network with Poisson distributions $P(\hat{\kappa}^{\pm})$. The intersections between the red line $R^{(t)} = R^{(t-1)}$ and the map (indicated in blue) determine the fixed points. Here $\underline{R}$ denotes the minimum non-trivial fixed point of the map. The map reaches its maximum $\hat{R}$ for $R = R^{\star}$. We denote with $\bar{R} > \underline{R}$ the point where $h(\bar{R}) = \underline{R}$. Panel (b) displays the map $R^{(t)} = h(R^{t-1})$ for different parameter p. In both panels the structural network is a Poisson network with average degree $c = 30$ and the distributions $\hat{P}_{\pm}(\hat{\kappa}_{\pm})$ are Poisson with average degrees $\langle \hat{\kappa}^{+} \rangle = c^{+} = 1.8$, $\langle \hat{\kappa}^{-} \rangle = c^{-} = 2.5$.

where $G_1(x)$, $G_0(x)$ and $G_0^{\pm}(x)$ are defined in Eq. (14). Starting from Eq. (37) and using the chain rule we get

$$\frac{dS^{(t)}}{dR^{(t-1)}} = \frac{\partial F_1}{\partial p_L^{(t-1)}} \frac{dp_L^{(t-1)}}{dR^{(t-1)}} + \frac{\partial F_1}{\partial S^{(t)}} \frac{dS^{(t)}}{dR^{(t-1)}}. \tag{40}$$

Thus,

$$\frac{dS^{(t)}}{dR^{(t-1)}} = \frac{\partial F_1}{\partial p_L^{(t-1)}} \frac{dp_L^{(t-1)}}{dR^{(t-1)}} \left(1 - \frac{\partial F_1}{\partial S^{(t)}}\right)^{-1}. \tag{41}$$

Similarly we can use the chain rule starting from Eq. (38) to express the derivative $dR^{(t)}/dR^{(t-1)}$, i.e.,

$$\frac{dR^{(t)}}{dR^{(t-1)}} = \frac{\partial F_2}{\partial p_L^{(t-1)}} \frac{dp_L^{(t-1)}}{dR^{(t-1)}} + \frac{\partial F_2}{\partial S^{(t)}} \frac{dS^{(t)}}{dR^{(t-1)}}. \tag{42}$$

Using Eq.(41) and the relation

$$\frac{\partial F_2}{\partial p_L^{(t-1)}} \frac{\partial F_1}{\partial S^{(t)}} = \frac{\partial F_1}{\partial p_L^{(t-1)}} \frac{\partial F_2}{\partial S^{(t)}}, \tag{43}$$

we obtain

$$\frac{dR^{(t)}}{dR^{(t-1)}} = \frac{\partial F_2}{\partial p_L^{(t-1)}} \frac{dp_L^{(t-1)}}{dR^{(t-1)}} \left(1 - \frac{\partial F_1}{\partial S^{(t)}}\right)^{-1}, \tag{44}$$

where

$$\begin{aligned} \frac{\partial F_1}{\partial p_L^{(t-1)}} &= S^{(t)} \langle k \rangle G_1 \left(1 - p_L^{(t-1)} S^{(t)}\right), \quad \frac{\partial F_1}{\partial S^{(t)}} = p_L^{(t)} \langle k \rangle G_1 \left(1 - p_L^{(t-1)} S^{(t)}\right), \\ \frac{\partial F_2}{\partial p_L^{(t-1)}} &= S^{(t)} G_1' \left(1 - p_L^{(t-1)} S^{(t)}\right), \quad \frac{\partial F_2}{\partial S^{(t)}} = p_L^{(t)} G_1' \left(1 - p_L^{(t-1)} S^{(t)}\right), \end{aligned} \tag{45}$$

and

$$\frac{dp_L^{(t-1)}}{dR^{(t-1)}} = p \left[G_0^-(1 - R^{(t-1)}) \left\langle \hat{\kappa}^+ \right\rangle G_1^+(1 - R^{(t-1)}) - \langle \hat{\kappa}^- \rangle G_1^-(1 - R^{(t-1)}) \left(1 - G_0^+(1 - R^{(t-1)})\right)\right].$$

Note that here $G_1'(x) = \sum_k k(k-1)x^{k-2}/\langle k \rangle$. From Eq.(44) and Eqs.(45) it follows that the derivative $dR^{(t)}/dR^{(t-1)}$ vanishes if and only if either $S^{(t)} = 0$ or $dp_L^{(t-1)}/dR^{(t-1)} = 0$. Consequently, the maximum of the map is determined by the condition $dp_L^{(t-1)}/dR^{(t-1)} = 0$. Let us now

consider the case in which the distributions $P(\hat{\kappa}^{\pm})$ are Poisson with average degree $c^{\pm}$. In this case $G_0^+(1-R^{(t-1)}) = G_1^+(1-R^{(t-1)}) = \exp(-c^+R^{(t-1)})$ and $G_0^-(1-R^{(t-1)}) = G_1^-(1-R^{(t-1)}) = \exp(-c^-R^{(t-1)})$ and hence

$$\frac{\partial p_L^{(t-1)}}{\partial R^{(t-1)}} = pe^{-c^-R^{(t-1)}}\left[-c^- + (c^+ + c^-)e^{-c^+R^{(t-1)}}\right]. \tag{46}$$

In this case there is only one singular value $R^{(t-1)} = R^{\star}$ at which $dp_L^{(t-1)}/dR^{(t-1)} = 0$ given by

$$R^{\star} = \frac{1}{c^+}\ln\left(\frac{c^+ + c^-}{c^-}\right). \tag{47}$$

It is straightforward to show that

$$\left.\frac{d^2p_L^{(t-1)}}{d(R^{(t-1)})^2}\right|_{R^{(t-1)}=R^{\star}} = -pe^{-(c^-+c^+)R^{\star}}(c^+ + c^-)c^+, \tag{48}$$

and, as long as $\hat{R} = h(R^{\star}) > 0$, it follows immediately that

$$\left.\frac{d^2R^{(t)}}{d(R^{(t-1)})^2}\right|_{R^{(t-1)}=R^{\star}} = \left.\frac{\partial F_2}{\partial p_L^{(t-1)}}\frac{\partial^2 p_L^{(t-1)}}{\partial(R^{(t-1)})^2}\left(1-\frac{\partial F_1}{\partial S^{(t)}}\right)^{-1}\right|_{R^{(t-1)}=R^{\star}} < 0. \tag{49}$$

Hence the scaling of the map close to the maximum is quadratic proving that the universality class of triadic percolation is the one of the logistic map as long as the structural and the regulatory degrees are uncorrelated and $P(\hat{\kappa}^{\pm})$ are Poisson distributions.

Stability of solutions and attractors Here we study the stability and the basin of attraction of the trivial solution $R^{(t)} = 0$ and the basin of attraction of the non-trivial attractor. In order to do we will investigate the major properties of the map function $R^{(t)} = h(R^{(t-1)})$ and we will make reference to the notation illustrated in Figure $S5(a)$.

For determining the basin of attraction of the zero solution $R^{(t)} = 0$ let us define $\underline{R}$ as the

smallest non-zero fixed point

$$\underline{R} \;=\; h(\underline{R}), \tag{50}$$

and let us consider $R^{(t-1)} < \underline{R}$. since $R^{(t)} < R^{(t-1)}$ for all t we derive that any initial condition $R^{(0)} < \underline{R}$ will eventually converge to the zero solution. Let us consider all the initial conditions $R^{(0)} > \bar{R}$ where $\bar{R}$ is the largest solution of the equation $\underline{R} = h(\bar{R})$. It is straightforward to see that also all these initial conditions will converge to the zero solution since after the first iteration of the map the problem can be reduced to the previous scenario.

Let us now establish the conditions that will ensure the stability of the non-trivial attractor. From the theory of coupled maps [1], we are guaranteed that the non-trivial attractor of the map will be stable as long as the interval $(\underline{R}, \bar{R})$ is mapped into itself or into one of its subsets by the map, i.e., as long as $\hat{R} = h(R^{\star}) < \bar{R}$.

4 Tuning the positive and negative regulatory interactions

Triadic percolation admits two limiting scenarios: the limit $c^{-} \to 0$ in which the model includes only positive regulatory interactions and is insensitive to negative regulations, and the limit $c^{+} \to \infty$ in which the role of positive regulatory interactions becomes negligible. In fact if $c^{-} \to 0$ then the condition that none of the negative regulators is active is always satisfied. On the contrary if $c_{+} \to \infty$ it becomes sure that at least one of the infinite positive regulators is active, so the role of positive regulators becomes negligible. In Fig. $S3$ and $S4$ we investigate the theoretically predicted orbit diagrams of triadic percolation for a Poisson structural network and for a scale-free

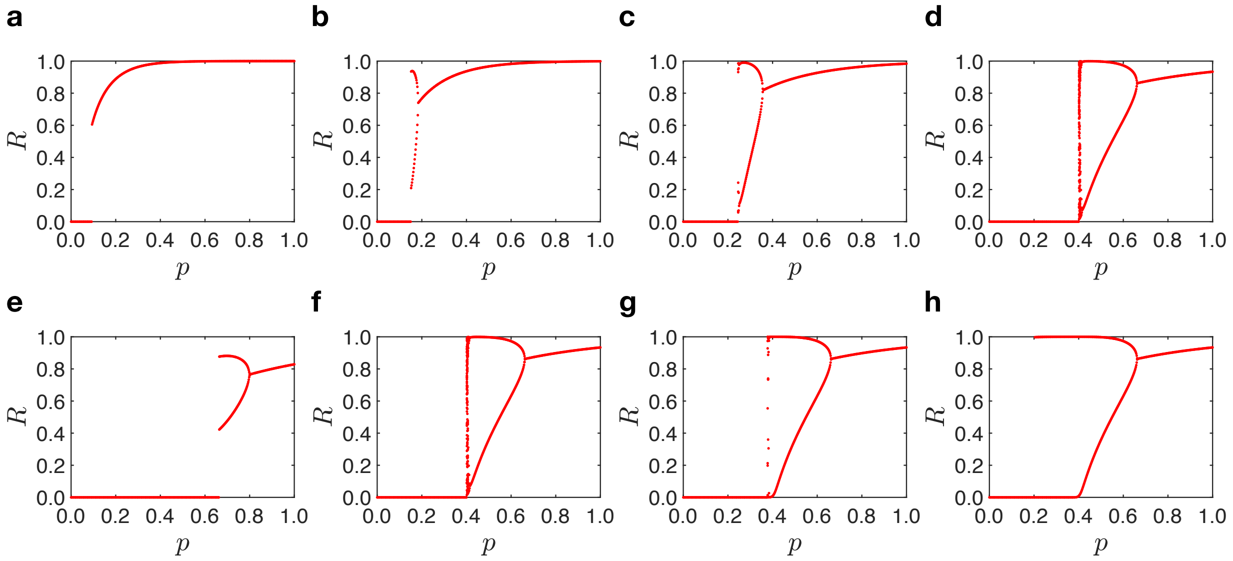

Figure S6: Theoretically obtained orbit diagrams for the Poisson structural network with average degree $c = 30$ and uncorrelated structural and regulatory degrees of the nodes. In the first row, $c^+ = 10$, from the left to the right we increase the c^- that $c^- = 1.0$ (a), $c^- = 1.5$ (b), $c^- = 2.0$ (c), $c^- = 2.5$ (d). In the second row, $c^- = 2.5$, from the left to the right we increase the c^+ that $c^+ = 1$ (e), $c^+ = 10$ (f), $c^+ = 1000$ (g), $c^+ = \infty$ (h). For all panels $c^\pm$ indicates the average degree of the Poisson distribution $\hat{P}_\pm(\hat{\kappa}^\pm)$. All figures are obtained by setting the initial condition $p_L^{(0)} = 0.1$.

structural network with structural degree uncorrelated with the regulatory degrees as a function of the average degrees c^+ and c^- of the Poisson distributions $\hat{P}_\pm(\hat{\kappa}^\pm)$. In absence of negative regulators, i.e., $c^- \to 0$, we observe a discontinuous hybrid transition in both cases (although displaying a smaller discontinuity for the scale-free structural network). For $c^+ = \infty$, we observe period-2 oscillations in the case of the Poisson structural network and a single stable solution in the scale-free case. In both cases, we observe chaos only in presence of both positive and negative regulatory interactions.

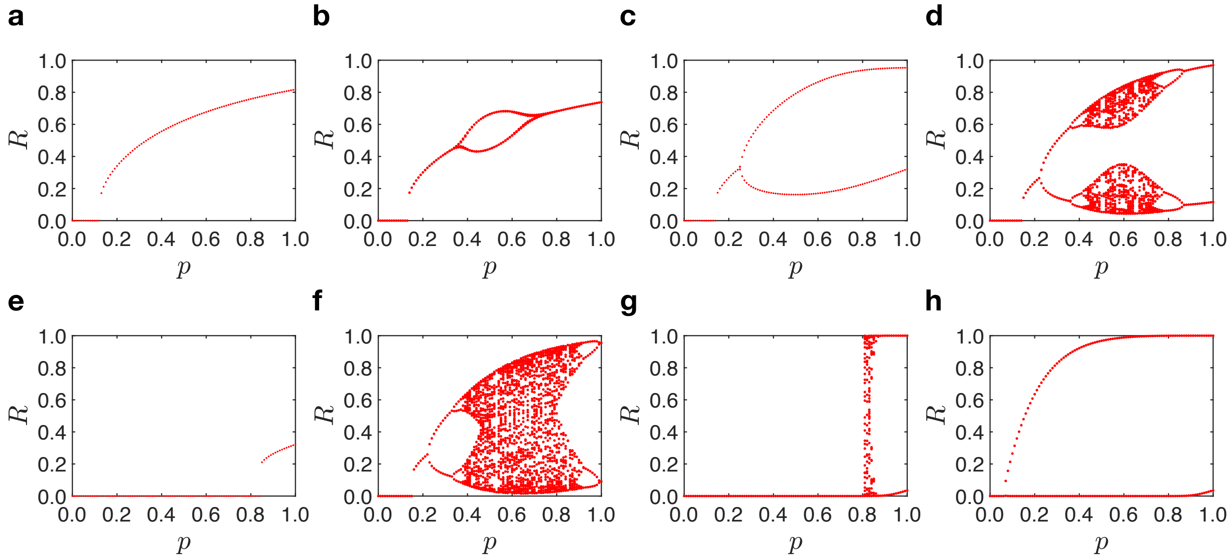

Figure S7: Theoretically obtained orbit diagrams of the scale-free structural network with minimum degree $m = 4$, power-law exponent $\gamma = 2.5$, maximum degree $K = 100$ and uncorrelated structural and regulatory degrees of the nodes. In the first row, $c^+ = 10$, from the left to the right we increase the c^- that $c^- = 1.5$ (a), $c^- = 1.9$ (b), $c^- = 2.3$ (c), $c^- = 2.7$ (d). In the second row, $c^- = 2.8$, from the left to the right we increase the c^+ that $c^+ = 1$ (e),$c^+ = 10$ (f), $c^+ = 1000$ (g), $c^+ = \infty$ (h). In all the panels $c^{\pm}$ indicate the average degree of the Poisson distribution $\hat{P}_{\pm}(\hat{\kappa}^{\pm})$. All figures are obtained by setting the initial condition to $p_L^{(0)} = 0.1$.

5 The role of the degree distribution of the structural network

In this paragraph we compare the phase diagram of triadic percolation for a scale-free structural network and for a Poisson structural network with the same average degree.

It is well known that for standard percolation the transition is always continuous and second

order with critical indices depending on the second moment of the degree distribution and therefore differing for scale-free networks with a power-law exponent $\gamma \leq 3$ and for Poisson networks. Moreover for the standard bond percolation, scale-free networks display a zero percolation threshold in the infinite network limit while Poisson networks with the same average degree have a finite percolation threshold [3]. This demonstrates the robustness of scale-free networks under random damage in the framework of the standard percolation theory. Indeed the hubs of scale-free networks, connecting a large set of nodes, keep the network connected also for an extensive entity of the damage of the links.

On the contrary, for interdependent percolation of multiplex networks the transition is discontinuous and scale-free networks are more fragile than Poisson networks with the same average degree [4]. This phenomenon is revealed by the percolation threshold of scale-free networks which is larger than the Poisson network with the same average degree and increasing with the power-law exponent γ. This phenomenon is due to the fact that hub nodes might be damaged easily if they are interdependent with nodes of small or average degree regardless of the state of their links within their layer.

In absence of negative regulatory interactions, the present model of triadic percolation displays a discontinuous transition as for interdependent networks. However structural scale-free networks remain more robust than structural Poisson networks.

In fact the percolation threshold at which the discontinuous transition occurs is smaller for scale-free networks than for Poisson networks with the same average degree (see Fig.$S8a$). This

is due to the fact that in triadic percolation the regulation acts directly on the links, and not on the nodes. Therefore the hubs can still play the role of keeping the structural network together also if the percolation transition becomes discontinuous.

When the average degree of the negative regulatory interactions in increased, the comparison of the phase diagram of the structural scale-free networks and the structural Poisson network with the same average degree continues to indicate a larger robustness of the structural scale-free network (see Fig. $S8b, c$ and d).

Indeed for larger values of average degree of negative regulatory interactions the structural Poisson network displays a period doubling and chaos of the order parameter while the structural scale-free networks display a discontinuous phase transition and small fluctuations of the order parameter (see Fig. $S8c$). For even larger values of the average degree of the negative regulatory interactions the structural Poisson network is dismantled for any possible value of p while the structural scale-free networks display period doubling and a route to chaos as a function of p (see Fig. $S8d$).

6 Comparison between the theory and the Monte Carlo simulations

In the main text, we have studied the period-doubling cascade and the route to chaos of triadic percolation as a function of the probability p that a link is active when all the regulatory interactions are satisfied. However, here we show that the period-doubling cascade and the route to chaos can also be observed for fixed value of p as a function of the average degree c^- of the Poisson

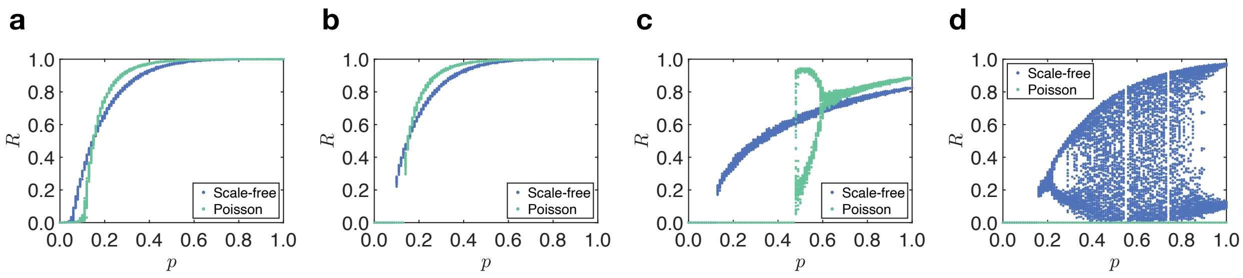

Figure S8: The comparison of orbit diagram between scale-free network and Poisson network with the same averaged degree. The structural scale-free graph has degree exponent $\gamma = 2.5$, minimum degree $m = 4$ and maximum degree $K = 100$. The regulatory network of both scale-free and Poisson structural network has a Poisson distribution with parameters (a) $c^+ = \infty$ and $c^- = 0$ (standard link percolation); (b) $c^+ = 10$ and $c^- = 0$; (c) $c^+ = 10$ and $c^- = 1.5$ and (d) $c^+ = 10$ and $c^- = 2.8$.

distribution $\hat{P}_-(\hat{\kappa}^-)$ in the case of uncorrelated structural and regulatory degrees of the nodes. In Fig. $S9$ and $S10$, we show the theoretically obtained orbit diagram as a function of c^- for a Poisson and for a scale-free network respectively and we compare the theoretical predictions with Monte Carlo simulations of triadic percolation for different values of c^- finding very good agreement.

In order to show furthermore the agreement between the theoretical expectation and the Monte Carlo simulations in Fig. $S11$ we compare the amplitude of the period-2 oscillations of the order parameter obtained with the Monte Carlo simulations with the predicted amplitude of the period-2 oscillations of the order parameter in a region of phase space where only period two oscillations are predicted. We find excellent agreement for both Poisson structural networks and scale-free structural networks with structural degree of the nodes uncorrelated with the regulatory

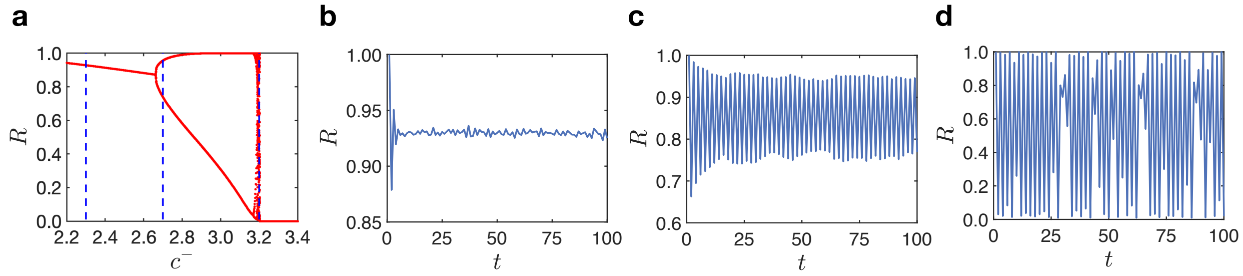

Figure S9: Theoretically obtained orbit diagram at fixed value $p = 0.8$ for the Poisson structural network, with average degree $c = 30$, uncorrelated structural and regulatory degree, Poisson distributed $\hat{\kappa}^{\pm}$ with average $\langle \hat{\kappa}^{+} \rangle = c^{+} = 10$ and $\langle \hat{\kappa}^{+} \rangle = c^{-}$. The three blue lines in panel (a) indicate $c^{-} = 2.3$, $c^{-} = 2.7$ and $c^{-} = 3.2$. The corresponding Monte Carlo simulations on networks of $N = 10^4$ are shown in panel (b),(c) and (d). All figures are obtained with an initial condition $p_L^{(0)} = 0.1$.

degrees of the nodes.

7 Further information about the real datasets investigated in the main text

We provide further information about the two datasets studied in Fig. 5 of the main text. In Supplementary Table 1 we provide the major structural properties of the networks and in Fig. $S12$ we report their degree distribution.

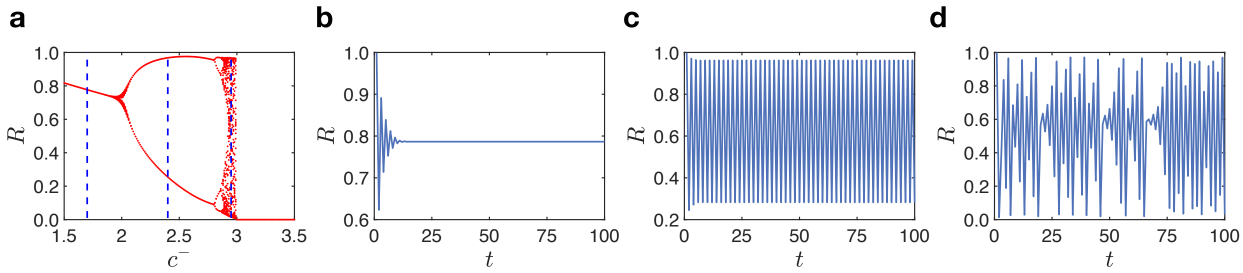

Figure S10: Theoretically obtained orbit diagram at fixed value $p = 1$ for the scale-free structural network, with minimum degree $m = 4$, maximum degree $K = 100$, power-law exponent $\gamma = 2.5$. The nodes have uncorrelated structural and regulatory degrees, and the distribution of $\hat{\kappa}^{\pm}$ is Poisson with average $\langle\hat{\kappa}^{+}\rangle = c^{+} = 10$ and $\langle\hat{\kappa}^{+}\rangle = c^{-}$. The three blue lines in panel (a) indicate $c^{-} = 1.7$, $c^{-} = 2.4$ and $c^{-} = 2.95$. The corresponding Monte Carlo simulations on a network of $N = 10^4$ nodes are shown in panel (b), (c) and (d). All figures are obtained with an initial condition $p_L^{(0)} = 0.1$.

8 Triadic percolation with time delays

In this section we provide the equations determining triadic percolation with time delays. In presence of time delays each regulatory link is assigned a time delay τ and Step 2 of the triadic percolation is modified by taking into account these time delays (see discussion in the main body of the paper). We have considered two variants of triadic percolation with time delays which depend on the choice of the probability distribution for time delays of regulatory links

- [Model 1] In Model 1 each structural link is regulated by links associated to the same time delay τ with the time delay τ being drawn from the distribution $\tilde{p}(\tau)$.

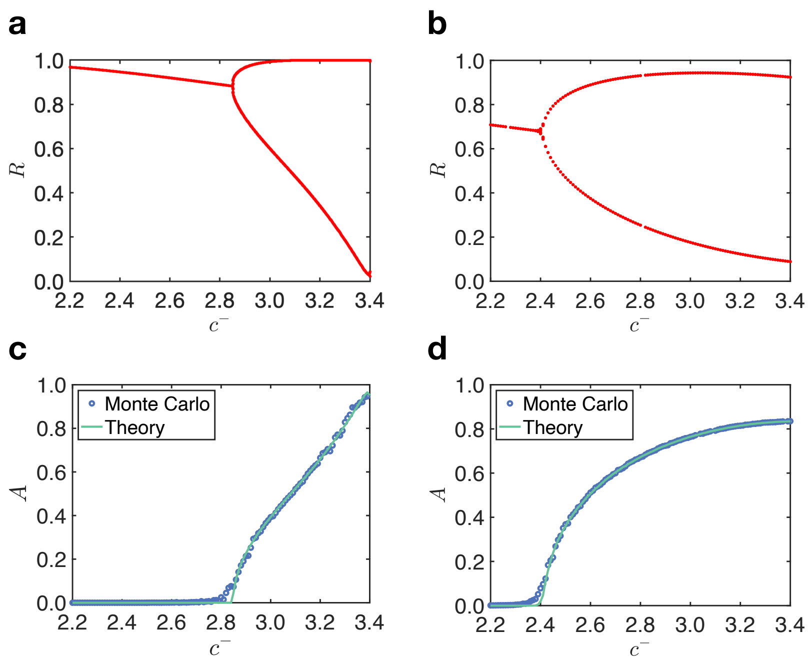

Figure S11: In panel (a) and (b) we show the theoretically obtained orbit diagram of triadic percolation on a Poisson network (a) and a scale-free network (b) for $p = 1$ as a function of c^- indicating the average degree of the Poisson distribution $\hat{P}_-(\hat{\kappa}^-)$. In panel (c) and (d), we plot the amplitude $A = \langle |R^{(t+1)} - R^{(t-1)}| \rangle$ of the period 2 oscillations of the order parameter obtained by Monte Carlo simulations (blue circles) of triadic percolation as a function of the average degree c^- (panel (c) refers to the same Poisson network as panel (a) and panel (d) refers to the same scale-free network as panel (b)). The theoretical prediction for the amplitude A are indicated with a green solid line in panels (c) and (d). The Poisson network (panels (a) and (c)) has average structural degree $c = 30$ and average degree of the Poisson distribution $\hat{P}_+(\hat{\kappa}^+)$ equal to $c^+ = 10$.The scale-free network (panels (b) and (d)) has minimum degree $m = 4$, power-law exponent $\gamma = 2.5$ and average degree of the Poisson distribution $\hat{P}_+(\hat{\kappa}^+)$ equal to $c^+ = 10$.The Monte Carlo simulations are conducted on networks of $N = 10^4$ nodes; the amplitude A is obtained by averaging over 10 network realizations.

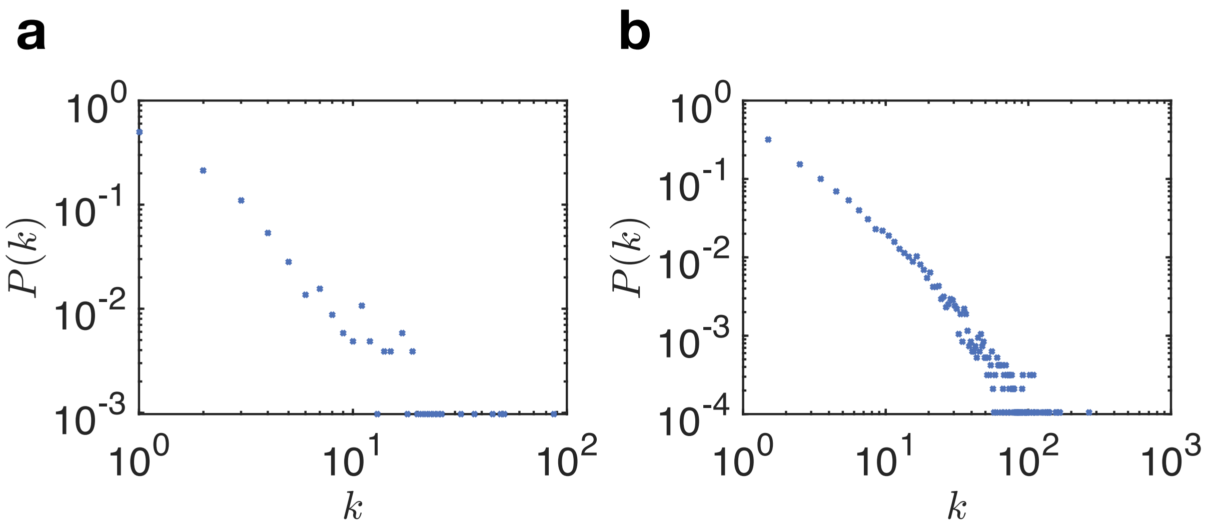

Figure S12: Degree distribution $P(k)$ of structural mouse brain network (a) and structural Human bio grid network (b) from [5].

- [Model 2] In Model 2 each regulatory link is associated to a time delay drawn independently from the distribution $\tilde{p}(\tau)$.

Both models lead to a route to chaos although this dynamics is in general not in the universality class of the logistic map.

Let us discuss how the equations determining the triadic percolation map are modified for Model 1 and Model 2 of triadic percolation with delay. We will focus on the case of uncorrelated structural and regulatory degree of the nodes with Poisson distributions $P(\hat{\kappa}^{\pm})$.

In this case the equations for triadic percolation with delay remain Eq.(11), Eq.(12) but Eq.(13) is modified to take into account the delay of the regulatory interactions. In particular

Network	C	k_{min}	k_{max}	N	L	R
Mouse Brain [5]	0	1	123	1029	1559	0.9592
Human bio grid [5]	0.1612	1	308	9436	31182	0.9642

Table S1: Structural properties of the real-world networks: the averaged clustering coefficient C, minimum degree k_{min}, maximum degree k_{max}, number of nodes N, number of links L and the fraction of node in the giant component R. Both networks are treated as undirected networks.

in Model 1 Eq. (13) is substituted by

$$p_L^{(t)} = p \sum_{\tau=1}^{d} \tilde{p}(\tau) e^{-c^- R^{(t+1-\tau)}} \left[1 - e^{-c^+ R^{(t+1-\tau)}}\right] \tag{51}$$

which takes into account that every structural link has all its regulatory links associated with the same delay τ where τ is draw from the distribution $\tilde{p}(\tau)$ for any structural link.

In the case in which Model 2 is considered, each regulatory interaction is associated to a delay τ with probability $\tilde{p}(\tau)$. Therefore among the $\hat{\kappa}^{\pm} = \hat{\kappa}$ positive or negative regulators of a link, the probability that n_i regulatory links are associated to a delay τ_i follows a multinomial distribution

$$\Pi(\{n_i\}_{i=1,2,\cdots,d} | \hat{\kappa}, \tilde{\mathbf{p}}) = \frac{\hat{\kappa}!}{\prod_{i=1}^{d} n_i!} \prod_{i=1}^{d} [\tilde{p}(\tau_i)]^{n_i}, \tag{52}$$

with $\tilde{\mathbf{p}} = (\tilde{p}(\tau_1), \tilde{p}(\tau_2), \ldots, \tilde{p}(\tau_d))$ and such that $\sum_{i=1}^{d} \tilde{p}(\tau_i) = 1$. Thus, Eq. (13) for triadic percolation is modified to

$$p_L^{(t)} = p \exp\left(-c^- \sum_{\tau_i} \tilde{p}(\tau_i) R^{(t+1-\tau_i)}\right) \left[1 - \exp\left(-c^+ \sum_{\tau_i} \tilde{p}(\tau_i) R^{(t+1-\tau_i)}\right)\right]. \tag{53}$$

For both cases above, if we consider one step delay, *i.e.*

$$\tilde{p}(\tau) = \delta_{\tau,1}, \tag{54}$$

Eq.(51) and Eq.(53) they both reduce to Eq.(13).

9 Regulation of the nodes's activity

In this work we have proposed the model of triadic percolation that demonstrates the important role of triadic interactions occurring in many real-world domains and whose role is related to the emergence of a time-dependent giant component. This model is motivated by the wide spread occurrence of triadic interactions in biological, chemical and climate applications.

However and interesting variation of the triadic percolation is to consider regulatory interactions that are regulating the activity of the nodes. In this case Step 2 of the algorithm can be modified as in the following.

Step $2''$ Each node is deactivated if at least one of the following conditions is met:

(a) each of its positive regulatory links is connected to a node that is inactive at Step 1;

(b) at least one of its negative regulator links is connected to a node that is active at Step 1.

(c) neither condition (a) or (b) is met but stochastic deactivation occurs with probability $q = 1 - p$.

In this case, assuming uncorrelated structural and regulatory degrees the dynamics will be

determined by the following equations

$$
\begin{aligned}
S^{(t)} &= p_L^{(t-1)}\left(1-G_1\left(1-S^{(t)}\right)\right), \\
R^{(t)} &= p_L^{(t-1)}\left(1-G_0\left(1-S^{(t)}\right)\right), \\
p_L^{(t)} &= pG_0^-\left(1-R^{(t)}\right)\left[1-G_0^+\left(1-R^{(t)}\right)\right].
\end{aligned}
\tag{55}
$$

Interesting this model displays period doubling and a route to chaos of the order parameter as well (see Figure $S13$). It can be directly shown, using an argument similar to the one used for triadic percolation, that the route to chaos observed in this model is in the same universality class of the logistic map as soon as $P(\hat{\kappa}^{\pm})$ indicating the distribution of the in-regulatory degree of the nodes with sign $\pm$ are Poisson distributed.

Supplementary References

1. Strogatz, S. H. *Nonlinear dynamics and chaos: with applications to physics, biology, chemistry, and engineering* (CRC press, 2018).

2. Feigenbaum, M. J. Quantitative universality for a class of nonlinear transformations. *Journal of statistical physics* **19**, 25–52 (1978).

3. Dorogovtsev, S. N., Goltsev, A. V. & Mendes, J. F. Critical phenomena in complex networks. *Reviews of Modern Physics* **80**, 1275 (2008).

4. Buldyrev, S. V., Parshani, R., Paul, G., Stanley, H. E. & Havlin, S. Catastrophic cascade of failures in interdependent networks. *Nature* **464**, 1025–1028 (2010).

5. Rossi, R. A. & Ahmed, N. K. The network data repository with interactive graph analytics and visualization. In *Proceedings of the Twenty-Ninth AAAI Conference on Artificial Intelligence* (2015). URL `http://networkrepository.com`.

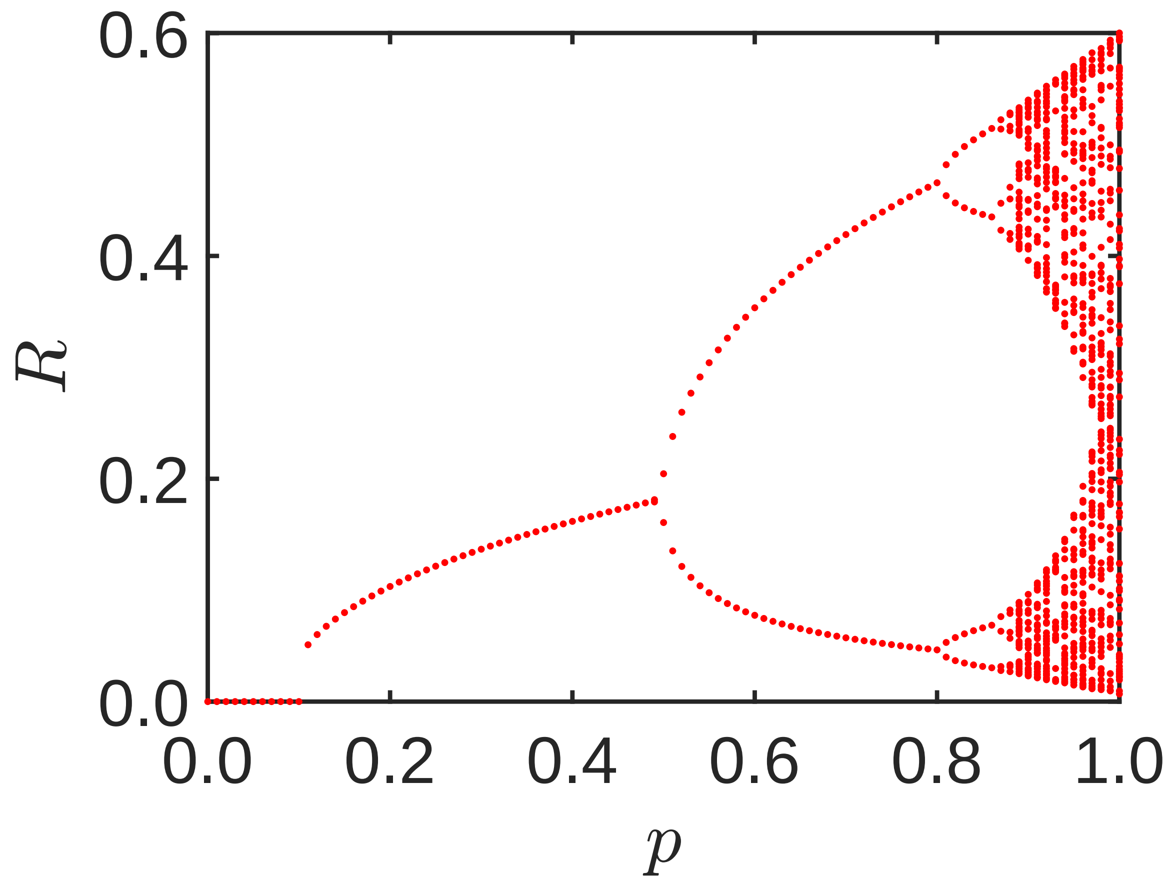

Figure S13: Phase diagram of percolation with regulation of the nodes's activity. The structural network is a Poisson network with an average degree $c = 30$ and the regulatory in-degree distributions $P(\hat{\kappa}^{\pm}$ is also Poisson distributed with average $c^{+} = 30$ and $c^{-} = 5.5$. The phase diagram also displays period doubling and a route to chaos in the universality class of the route the logistic map.
