# Supplementary material for: The dynamic nature of percolation on networks with triadic interactions
Source: Nat Commun. 2023 Mar 10;14:1308. doi: 10.1038/s41467-023-37019-5 (PMC9998640; doi:10.1038/s41467-023-37019-5)
Supplement: Supplementary file 2 — Description of Additional Supplementary Files [file 41467_2023_37019_MOESM2_ESM.pdf]

<u>Description of Additional Supplementary Files</u>

Supplementary Movie: Movie of triadic percolation.The structural network is a Poisson networks of 20 nodes and average degree $c=4$ and $p=1$.The regulatory network has Poisson positive and negative regulatory degree distribution $c^+=4$ and $c^_=1$. The coordinates of nodes in the movie are randomly assigned. Active nodes are highlighted in orange and active links are show in blue (inactive links are not shown).
