# Supplementary figures and images for: The dynamic nature of percolation on networks with triadic interactions
Source: Nat Commun. 2023 Mar 10;14:1308. doi: 10.1038/s41467-023-37019-5 (PMC9998640; doi:10.1038/s41467-023-37019-5)

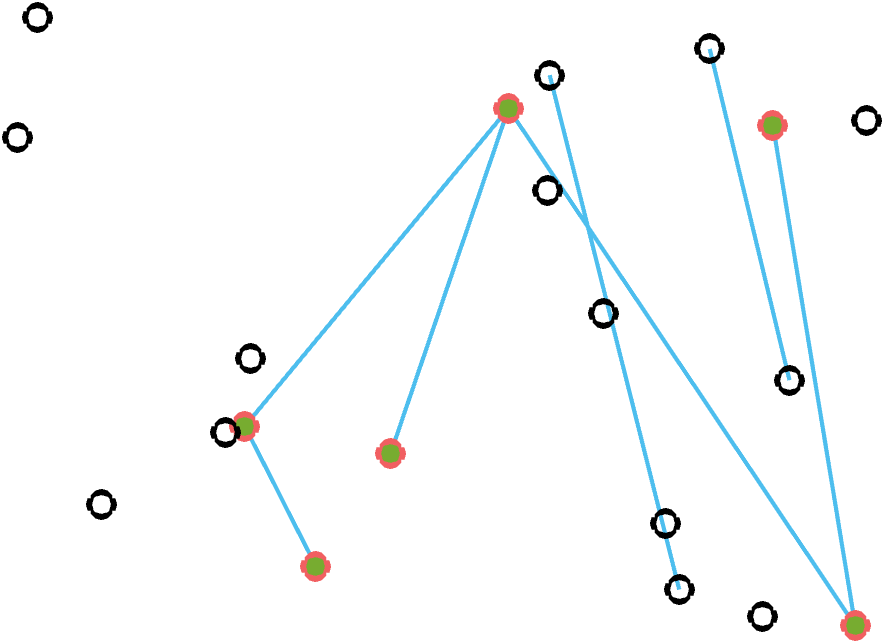

Supplement: Supplementary file 3 — Supplementary Movie [file 41467_2023_37019_MOESM3_ESM.gif]
